# Transcriptomic analysis of rat brain response to alternating current electrical stimulation: unveiling insights via single‐nucleus RNA sequencing

**DOI:** 10.1002/mco2.514

**Published:** 2024-03-15

**Authors:** Yan Wang, Yongchao Ma, Qiuling Zhong, Bing Song, Qian Liu

**Affiliations:** ^1^ Institute of Biomedical and Health Engineering Shenzhen Institutes of Advanced Technology Chinese Academy of Sciences Shenzhen China

**Keywords:** cell‐type‐specific response, gene expression change, intracranial alternating current stimulation, neuron, single‐nucleus RNA sequencing

## Abstract

Electrical brain stimulation (EBS) has gained popularity for laboratory and clinical applications. However, comprehensive characterization of cellular diversity and gene expression changes induced by EBS remains limited, particularly with respect to specific brain regions and stimulation sites. Here, we presented the initial single‐nucleus RNA sequencing profiles of rat cortex, hippocampus, and thalamus subjected to intracranial alternating current stimulation (iACS) at 40 Hz. The results demonstrated an increased number of neurons in all three regions in response to iACS. Interestingly, less than 0.1% of host gene expression in neurons was significantly altered by iACS. In addition, we identified *Rgs9*, a known negative regulator of dopaminergic signaling, as a unique downregulated gene in neurons. Unilateral iACS produced a more focused local effect in attenuating the proportion of *Rgs9*+ neurons in the ipsilateral compared to bilateral iACS treatment. The results suggested that unilateral iACS at 40 Hz was an efficient approach to increase the number of neurons and downregulate *Rgs9* gene expression without affecting other cell types or genes in the brain. Our study presented the direct evidence that EBS could boost cerebral neurogenesis and enhance neuronal sensitization to dopaminergic drugs and agonists, through its downregulatory effect on *Rgs9* in neurons.

## Graphical abstract

We provide here the first single‐nucleus RNA sequencing profiles of the rat cortex, hippocampus, and thalamus under the intracranial alternating current stimulation (iACS). The findings would support as direct evidence that transcranial, intracranial, or deep brain iACS may increase neuronal number and sensitization to dopaminergic or dopaminergic agonists, improve analgesic effects, and prolong drug tolerance through its unique down‐regulatory effect on Rgs9 in neurons.

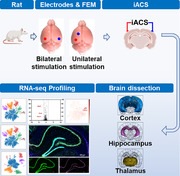

## 1 INTRODUCTION

The use of electrical brain stimulation (EBS) as a therapeutic modality for treatment of brain disorders has evolved significantly, with recent developments pushing the boundaries of our understanding and treatment capabilities.^1^, ^2^, ^3^, ^4^, ^5^, ^6^ The principle of this approach is to use precisely applied electrical currents to modulate neuronal activity, such as neuronal membrane stability and action potential, ion and neurotransmitter fluxes, intracellular pathway activation, etc., to treat Parkinson's disease (PD), Alzheimer's disease (AD), and other central nervous system (CNS) disorders.^6^, ^7^, ^8^, ^9^, ^10^, ^11^, ^12^, ^13^, ^14^

Take treatment for AD as an example, studies have suggested that the use of 40 Hz flicker stimulation can modulate abnormal gamma oscillations in the brain waves of AD model mice to some extent, thereby improving impaired learning and memory functions.^15^, ^16^, ^17^ However, the latest research refutes this finding, claiming that flicker stimulation cannot regulate gamma oscillations in brain waves, nor can it reduce excessive Aβ deposition in the brains of AD mice or ameliorate AD symptoms.^18^ Compared to indirect neural stimulation by visual flicker, EBS may more directly modulate brain activity and pathological processes. Among the EBS approaches, transcranial alternating current (AC) stimulation (tACS) is considered the optimal therapeutic strategy to achieve direct brain stimulation, non‐invasively.^19^ However, studies confirm that 75%–85% of the main therapeutic current of transcranial stimulation is shielded by the scalp and skull.^20^ In contrast, intracranial AC stimulation (iACS), which is achieved by implanting electrodes within the cranial bone, bypasses the shielding effects of the scalp and skull.^21^ Without penetrating the brain parenchyma, iACS can deliver the full intensity stimulating currents directly to the brain to achieve therapeutic effects. Therefore, when tACS is the optimal clinical strategy, iACS, which reflects the modulation effects on brain, is more suitable to elucidate the modulating mechanisms of EBS on healthy brain and neural disorders.

Our previous studies have demonstrated the effectiveness of iACS in enhancing neurogenesis and modulating microglial activation in AD mice. Furthermore, we also observed an augmentation in neuronal differentiation of neural stem cells by in vitro electric stimulation.^21^, ^22^, ^23^ These preliminary studies inspired further exploration on the precise cellular and genetic mechanisms by EBS, an area of research that, to our knowledge, remains largely unexplored.

Here, we provided the first single‐nucleus RNA sequencing (snRNA‐seq) profiles of the rat brain with iACS, separately profiling 285,347 single‐nucleus transcriptomes from the cortex, hippocampus, and thalamus of eight rats, aiming to profile distinct responding cell clusters and their gene expression patterns following the specific 40 Hz iACS trials. Our objective was to delineate the specific cell types and genes that response to iACS under normal physiological conditions, prior to any administration for brain diseases. The results would provide essential early insights into the safety of neural cells and genes within the brain under the 40 Hz iACS neural modulation and identify potential cellular targets. This research would be instrumental in shaping strategies for administering neural modulation to both individuals in health and brain disease conditions, such as AD, PD, stroke, etc.

## 2 RESULTS

### 2.1 Intracranial alternating current stimulation schemes and in‐brain‐derived electric field distribution

**FIGURE 1:**
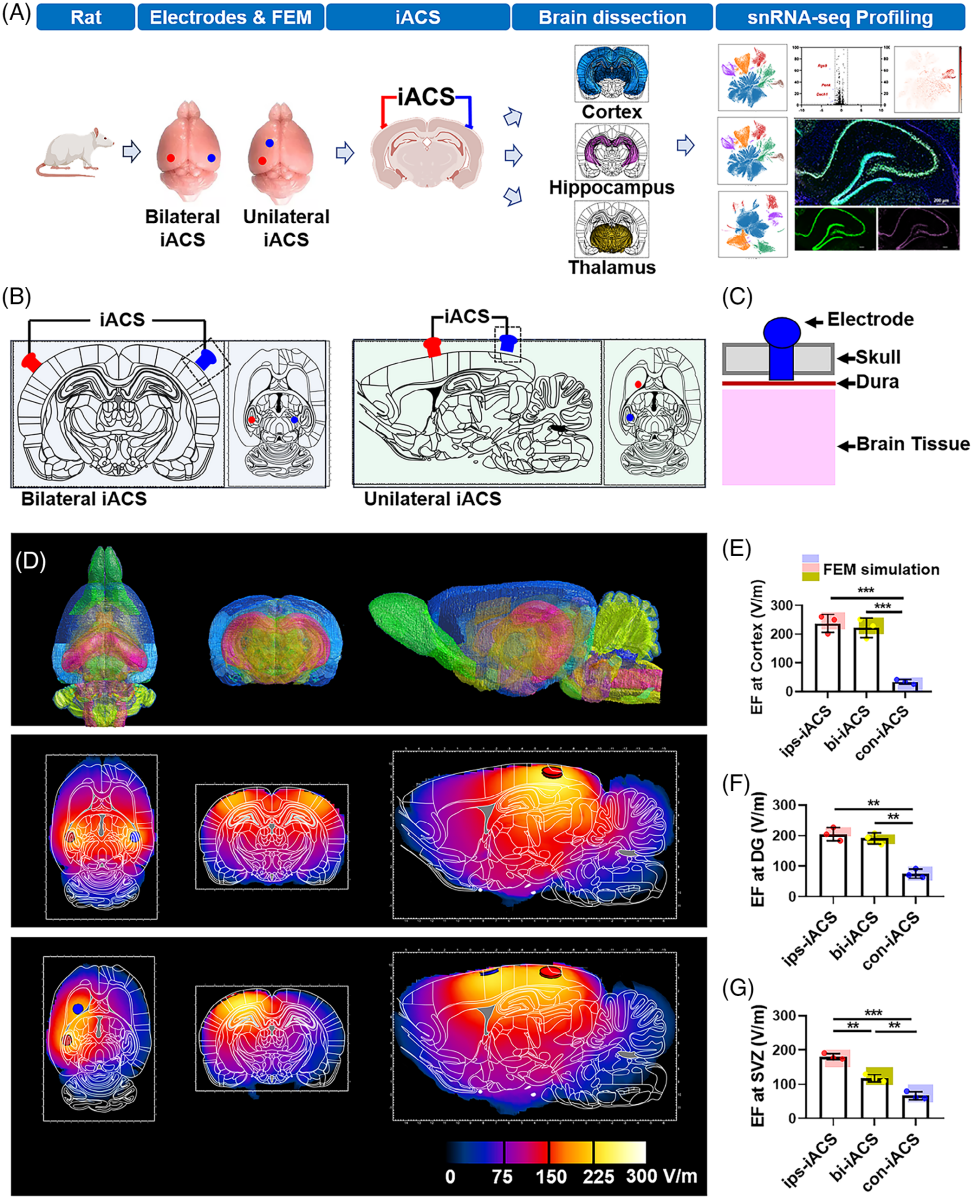
Research design of intracranial alternating current stimulation (iACS) and derived in‐brain electric field (EF). (A) Research design. (B) iACS scheme illustration: bilateral iACS (bi‐iACS, left) and unilateral iACS (right). (C) The iACS electrode implanted in skull for stimulating current delivery. (D) The 3D brain modeling and finite element method (FEM) simulation of iACS‐derived in‐brain EF distribution. The upper row was the 3D brain modeling, build up based on a volumetric atlas (including 118 brain structures) offering comprehensive anatomical delineations of the male Sprague–Dawley rat brain; the middle row was the FEM simulation of the derived EF distribution by the bilateral iACS, at 40 Hz, 250 µA. The lower row was the FEM simulation of the derived EF distribution by the unilateral iACS for the left hemisphere, at 40 Hz, 250 µA. (E) The measurement of derived EF at the ipsilateral (ips‐iACS)/contralateral (con‐iACS) forebrain cortex under the unilateral iACS, or at the forebrain cortex under the bi‐iACS. (F) The measurement of derived EF at the dentate gyrus (DG) of the hippocampus under the ips‐iACS, bi‐iACS, and con‐iACS. (G) The measurement of derived EF at the sub‐ventricular zone (SVZ) of the thalamus under the ips‐iACS, bi‐iACS, and con‐iACS. The blue, pink, and yellow rectangles illustrated the ranges of the FEM simulated EF magnitudes under ips‐iACS, bi‐iACS, and con‐iACS from (D). The measured EF magnitudes were shown as mean ± standard deviation (SD), ^***^ *p* < 0.001 and ^**^ *p* < 0.01 were considered as significantly different between groups. *n* = 3 rats for each group.

**FIGURE 2:**
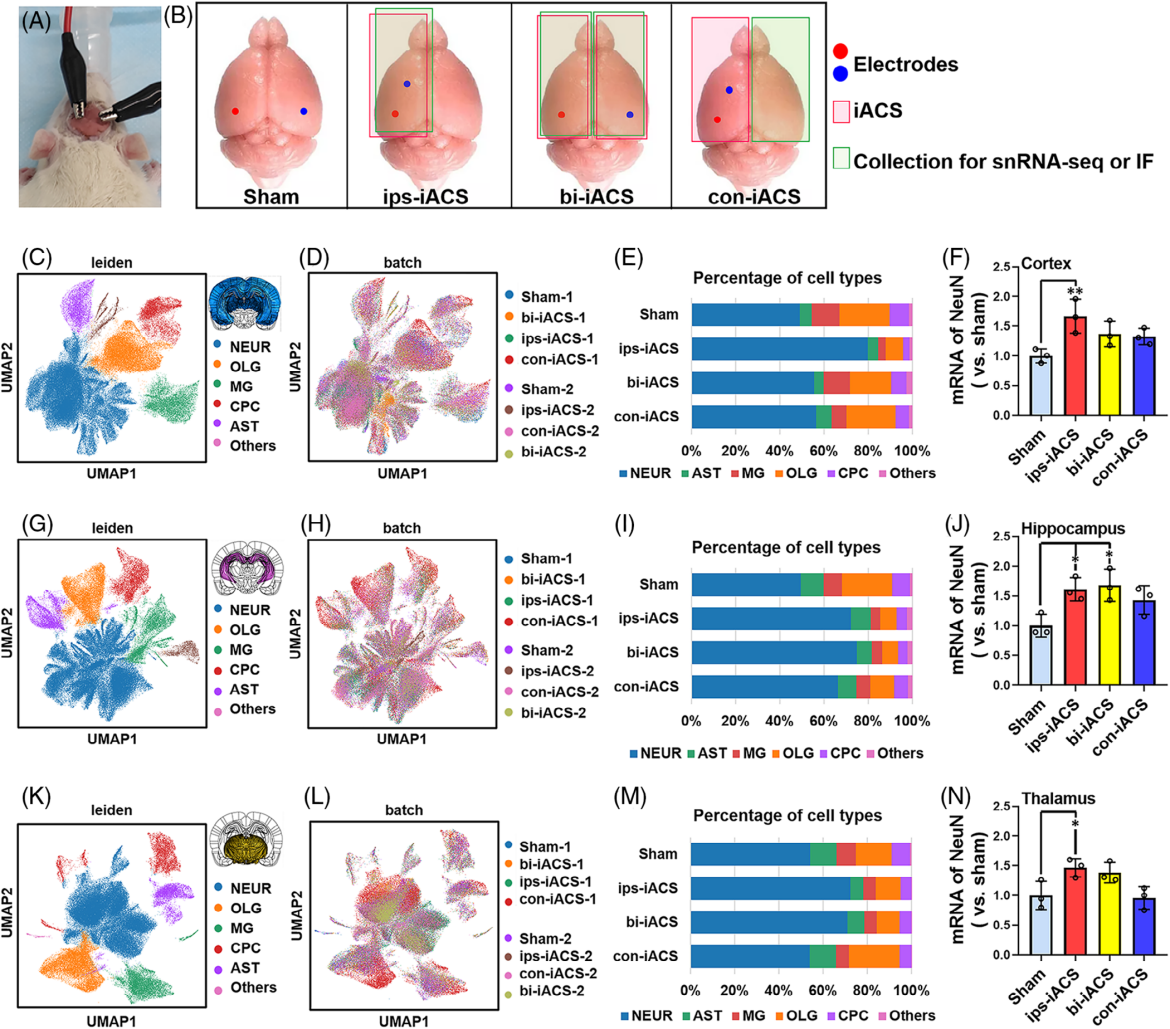
The single‐nucleus RNA sequencing (snRNA‐seq) profiling of the cortex, hippocampus, and thalamus of intracranial alternating current stimulation (iACS)‐treated rats. (A) Photograph of a rat underling the iACS treatment. (B) Grouping illustration for electrode implantation, iACS and tissue collection. Uniform manifold approximation and projection (UMAP) embedding of analyzed transcriptomes from the rat cortex (C and D), hippocampus (G and H), and thalamus (K and L) tissue annotated by cell type as well as treatment distribution. *n* = 2 rats for each group for snRNA‐seq. Distribution and cell numbers of identified cell types, for the cortex (E), hippocampus (I), and thalamus (M). The mRNA expression of NeuN in the iACS‐treated cortex (F), hippocampus (J), and thalamus (N) by real‐time PCR. The mRNA expression of NeuN by real‐time PCR were shown as mean ± standard deviation (SD), ^***^ *p* < 0.001, ^**^ *p* < 0.01, and ^*^ *p* < 0.05 were considered as significantly different between each iACS and sham. *n* = 3 rats for each group for the real‐time PCR assay.

We have designed the study to use randomly grouped rats, subjecting to bilateral or unilateral iACS trials. The brain regions of cortex, hippocampus, and thalamus from each experimental group of rats were, respectively, collected for snRNA‐seq, gene and protein expression analyses (Figure 1A). The electrode implantation was designed for either bilateral or unilateral iACS treatment as shown in Figures 1B and 2B. To intracranially deliver the current into brain, the electrodes were drilled in the skull without penetrating the cerebral tissue. The end of electrode was precisely positioned to contact the dura, thereby delivering the electrical current, as depicted in Figure 1B,C. For the sham and bilateral iACS (bi‐iACS) groups, the paired electrodes were set symmetrically on each hemisphere (left lane in Figure 1B; sham and bi‐iACS in Figure 2B) to deliver the fake or iACS current bilaterally. For unilateral iACS stimulation, the paired electrodes were set on the left hemisphere (right lane in Figure 1B) for ipsilateral and contralateral iACS, shortened as ips‐iACS and con‐iACS in Figure 2B. The iACS trial started 24 h after the electrode implantation surgery. The sinusoidal current was delivered intracranially at 40 Hz, 250 µA, 1 h per day for 7 days (Figures 2A and S1A,B). The rats kept in health status during and after the iACS treatment each day, according to the daily records of body weight (Figure S1C) and neurological severity score (NSS) assessment (Figure S1D), indicating no hazards to health or neurological functions by iACS. We also performed hematoxylin and eosin immunocytochemistry with the brains at the end of the experiment, showing no significant tissue damage the iACS treatment (Figure S1E).

To quantify the coverage of iACS in deeper brain, we performed the finite element method (FEM) simulation and measured the in‐brain‐derived electric field (EF) in rat brain with unilateral or bilateral iACS. With FEM (Figure 1D), the simulation showed an obviously derived in‐brain EF when either bilateral or unilateral iACS were administrated on the 3D rat brain model (upper row in Figure 1D). The peak magnitude of EF was estimated at ∼300 V/m, surrounding the electrode areas, attenuating deeper into the brain. Specifically, when bilateral iACS was performed, the FEM simulation plotted a symmetric distribution of EF on both hemispheres. For the cortex, dentate gyrus (DG) of the hippocampus and sub‐ventricular zone (SVZ) of the thalamus, the EF magnitude was read at ranges of 200–250 V/m, 170–200 V/m, and 100–150 V/m, respectively (middle row in Figure 1D). While, the unilateral iACS simulation plotted an even higher peak of EF, distributing mainly at the ipsilateral hemisphere (ips‐iACS). The peaks of EF were read as 225–275 V/m EF in the cortex, 200–225 V/m in the DG, and 150–200 V/m in the SVZ (left hemisphere in the bottom row in Figure 1D). While for the contralateral hemisphere (con‐iACS), it plotted a significantly lower EF, compared to the relative ipsilateral plot. The estimated peaks of EF were read as 0–50 V/m in the cortex and 50–100 V/m in both the DG and SVZ (right hemisphere in the bottom row in Figure 1D).

For the measurement in iACS‐treated rat brain, the real‐time EEG revealed a marked peak in gamma oscillation (within the 35−60 Hz range) following the 40 Hz iACS administration, irrespective of unilateral or bilateral iACS scheme (Figure S2). On the other hand, the in‐brain‐derived EF magnitude was detected varying upon the specific iACS scheme was employed. Specifically, the oscilloscope measurement demonstrated that ips‐iACS generated the highest EF in the cortex (237.3 ± 31.6 V/m), the DG (204.9 ± 21.8 V/m), and the SVZ (180.7 ± 8.9 V/m). These values were higher compared to those obtained from the bi‐iACS group, which recorded 222.0 ± 33.7 V/m in the cortex, 190.8 ± 18.4 V/m in the DG, and 117.8 ± 10.8 V/m in the SVZ. The con‐iACS showed considerably lower EF values, with 33.8 ± 8.2 V/m in the cortex, 75.4 ± 14.6 V/m in the DG, and 66.7 ± 11.2 V/m in the SVZ. Notably, these in‐brain EF measurements were consistent with the FEM simulation plots (Figure 1E–G).

The results from both FEM simulation and in‐brain EF measurement indicated that iACS could deliver the 40 Hz signal at 250 µA into deeper brain while maintaining the full frequency. However, there was a progressive decrease in the intensity of EF as the distance increased from the surface to deeper brain. Furthermore, unilateral iACS resulted a focused local impact within the ipsilateral hemisphere. While, bilateral iACS encompassed a more extensive area, affecting both hemispheres, but with a less concentrated intensity of EF.

### 2.2 snRNA‐seq profiling of cortex, hippocampus, and thalamus of the iACS rats

Following the 7‐day iACS trial, the rats were sacrificed for brain region snRNA‐seq, real‐time PCR, and immunofluorescence analyses. The brain collections were divided into four groups for sample labeling: sham (with fake iACS), ips‐iACS (the ipsilateral hemisphere with unilateral iACS), bi‐iACS (with bilateral iACS), and con‐iACS (the contralateral hemisphere with unilateral iACS) (Figure 2A,B).

Here, we separately profiled the cortex, hippocampus, and thalamus of the above four‐group brain hemisphere for specific‐region snRNA‐seq (*n* = 2 rats in each group for snRNA‐seq). In total, 285,347 snRNA‐seq profiles, including 108,996 cells in the cortex (Figure 2C,D), 88,592 cells in the hippocampus (Figure 2G,H), and 87,759 cells in the thalamus were analyzed as shown in Figure 2K,L.

To classify the major cell types in specific regions of the iACS rat brain, we clustered all cells jointly across the eight individual rat cortex, hippocampus, or thalamus, producing six transcriptionally distinct cell‐type clusters for each of the three brain regions with highly consistent expression patterns across the individual iACS rat brains. We identified and annotated the major cell types of the cortex (Figure 2C), hippocampus (Figure 2G), and thalamus (Figure 2K) by interrogating the expression patterns of known gene markers,^23^, ^24^ neurons (marked by *Syt1* and *NeuN*), oligodendrocytes (marked by *Cldn11*), microglia (marked by *Tmem176b*), choroid plexus cells (marked by *Vcan*), astrocytes (marked by *Gja1*), and others (Figures S3A–C, S5A–C, and S7A–C). We then tracked and annotated each single cell from all clusters of each individual rat with the specific iACS scheme (Figure 2D for the cortex, Figure 2H for the hippocampus, and Figure 2L for the thalamus). We used these tracking and annotations to quantify the ratios of the cell type in the cortex, hippocampus, and thalamus, to identify the cell‐type‐specific response to iACS, to assess differences in region‐specific cellular responses between unilateral and bilateral iACS, and to characterize the specificity of iACS‐modulated gene expression.

### 2.3 Neuronal populations were boosted by iACS

To dissect cell‐type heterogeneity under iACS across different regions of brain, we separately quantified the percentages of each cell‐type category from the cortex, hippocampus, and thalamus in the four groups of sham, ips‐iACS, bi‐iACS, and con‐iACS. Incorporating the results from the snRNA‐seq, real‐time PCR, and immunofluorescence analyses, the comparison between groups demonstrated the following cell‐type‐composition‐specific responses to iACS treatments with brain region specificity.

*In the cortex*, the region most closely exposed to iACS current, the 7‐day iACS trial increased the percentage of neurons with either ips‐iACS (increased by 63.1%), bi‐iACS (by 13.3%), or con‐iACS (by 9.3%) treatment, compared with those in sham, according to the snRNA‐seq (Figure 2E).

In consistent with this result of snRNA‐seq, the mRNA expression of *NeuN* (a neuronal marker), according to the real‐time PCR, were found to be upregulated following treatments with ips‐iACS by 66.8% (*p* = 0.0089, *n* = 3 rats for each group), bi‐iACS by 36.8% (*p* = 0.1271, *n* = 3), and con‐iACS by 32.6% (*p* = 0.1844, *n* = 3), in comparison to sham (Figure 2F). The ips‐iACS emerged as the most effective iACS scheme for increasing the neuronal population in the cortex. Alongside this increase, there was a noted decrease in the percentages of oligodendrocytes, microglia, and choroid plexus cells when ips‐iACS was applied. However, the significant trend of decrease was not detected in either the bi‐iACS or con‐iACS group. On the other hand, astrocytes exhibited irregular changes in response to the iACS treatments, compared to sham (Figure 2E).

**FIGURE 3:**
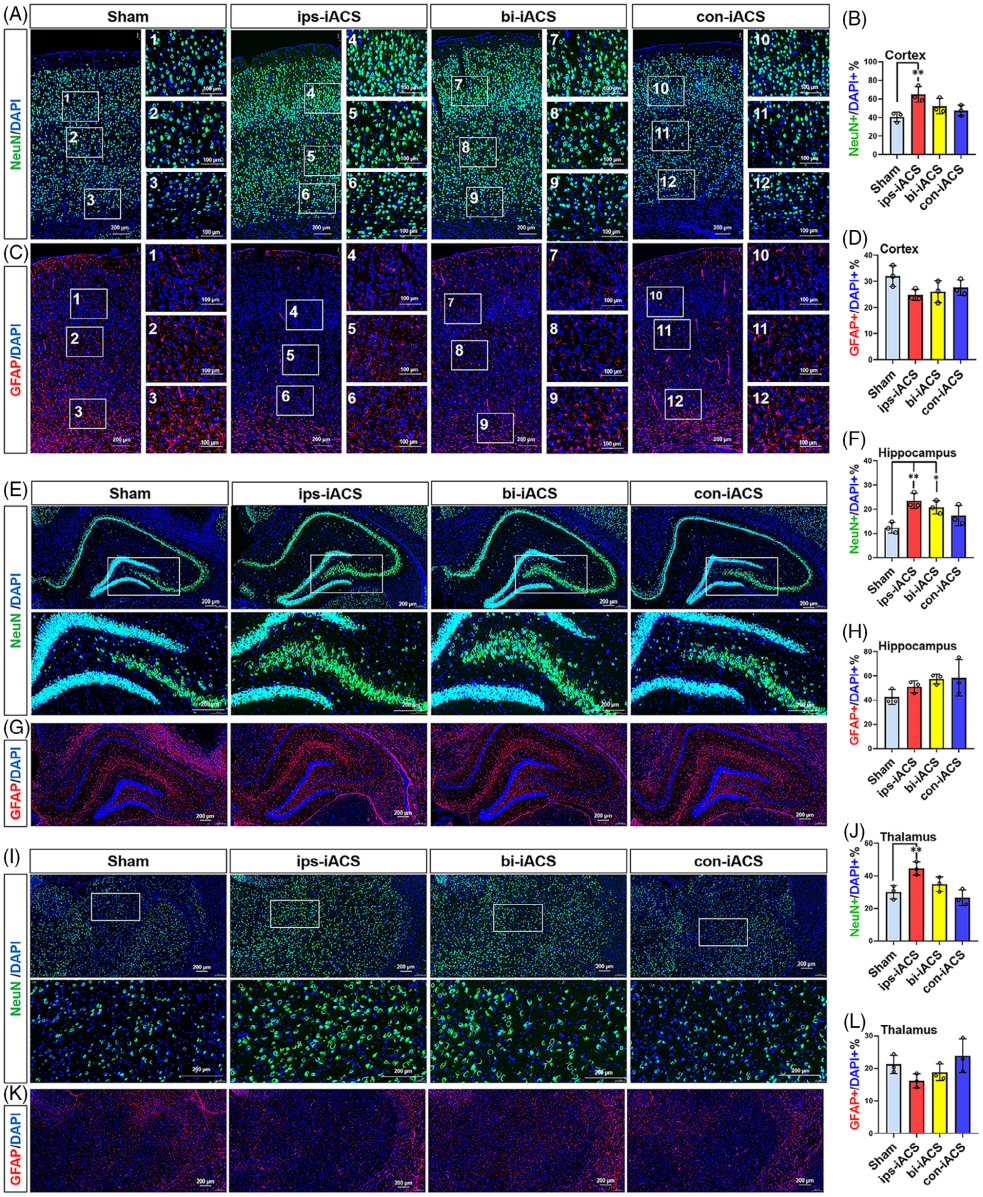
The immunofluorescence of neurons and glia in cortex, hippocampus, and thalamus of intracranial alternating current stimulation (iACS) rat. The NeuN+ neurons in the cortex (A), hippocampus (E), and thalamus (I). Quantification of NeuN+ neuron count percentage in the cortex (B), hippocampus (F), and thalamus (J). GFAP+ cells in the cortex (C), hippocampus (G), and thalamus (K). Quantification of GFAP glia count percentage in the cortex (D), hippocampus (H), and thalamus (L). The nuclei were labeled with 4',6‐diamidino‐2‐phenylindole (DAPI) staining. The percentages of NeuN+/DAPI+ and GFAP+/DAPI+ were shown as mean ± standard deviation (SD), ^***^ *p* < 0.001, ^**^ *p* < 0.01, and ^*^ *p* < 0.05 were considered as significantly different between each of the three iACS groups and sham. *n* = 3 rats for each group. Score bars: 200 or 100 µm as shown in each image.

We then applied immunofluorescence to verify that ips‐iACS led to the most pronounced increase in NeuN+ (encoding protein of *NeuN*) neurons in the cortex, as depicted in Figure 3A,B. The zoomed‐in images in Figure 3A1–12, displayed the expression patterns of NeuN in the experimental groups, with a focus on the different layers of the cortex. The quantified proportion of NeuN+ cells demonstrated a significant rise in NeuN+ neurons with both ips‐iACS and bi‐iACS. Specifically, the proportion of NeuN+ neurons increased from 40.55% in sham to 65.01% with ips‐iACS (*p* = 0.0072, *n* = 3 rats for each group). However, the increases to either 52.39% with bi‐iACS (*p* = 0.1682, *n* = 3) or 47.54% (*p* = 0.5137, *n* = 3) with con‐iACS did not yield statistical significance when compared to sham, indicating a less promising effect. On the other hand, the quantification of GFAP+ glial cells revealed no significant changes following any of the three iACS treatments compared to sham (Figure 3C,D). The results aligned with the snRNA‐seq profiling (Figure 2E) and the *NeuN* expressions determined through real‐time PCR (Figure 2F).

Combining the results from snRNA‐seq, real‐time PCR, and immunofluorescence analyses using the cortex samples, ips‐iACS was the most effective in increasing neurons in the cortex. While, neither significant nor regular change was detected in glial cells with any scheme of iACS.

*In the hippocampus*, neurons continued to be the most responsive cell type to the three iACS schemes. According to the snRNA‐seq profiling, the increase in neurons was quantified as follows: ips‐iACS led to a 46.1% increase, bi‐iACS resulted in a 51.7% increase, and con‐iACS achieved a 34.5% increase in hippocampal neurons compared to sham (Figure 2I). Notably, bi‐iACS was more effective in increasing neuron percentage in the hippocampus than that with ips‐iACS.

The real‐time PCR analysis confirmed the consistent trends of the neuron increase with the *NeuN* expression in the hippocampus following the iACS treatments. Specifically, compared to sham, the expression of *NeuN* was increased by 61.0% (*p* = 0.0265, *n* = 3 rats for each group) with ips‐iACS, by 67.9% (*p* = 0.0155, *n* = 3) with bi‐iACS, and by 42.9% (*p* = 0.1132) with con‐iACS, as shown in Figure 2J. As for the other cell types in the hippocampus, oligodendrocytes were detected decreased by the iACS treatments. While astrocytes and microglia demonstrated no significant change across the experimental groups (Figure 2I).

Immunofluorescence revealed that all three iACS groups exhibited an increase in NeuN+ neurons within the DG and Cornu Ammonis 4 (CA4) areas of the hippocampus. Notably, the percentage of NeuN+ neurons rose from 12.45% in sham to 23.52% (*p* = 0.0063, *n* = 3 rats for each group) with ips‐iACS and to 20.84% (*p* = 0.0272, *n* = 3) with bi‐iACS, reaching statistical significance. Increase was also observed with con‐iACS to 17.42% (*p* = 0.1955, *n* = 3); however, the change did not show statistical significance, as shown in Figure 3E,F. The zoomed‐in images distinctly highlighted the difference in the number of NeuN+ cells among the iACS treated and sham within the DG and CA4 areas of the hippocampus (Figure 3E). In contrast, GFAP+ glial cells did not show any significant alterations following any scheme of iACS compared to sham in the hippocampus, as shown in Figure 3G,H.

The snRNA‐seq, real‐time PCR, and immunofluorescence analyses indicated that ips‐iACS and bi‐iACS were highly effective in increasing neuronal numbers in the hippocampus. While con‐iACS did not exhibit a significant effect on neuronal increasement. Additionally, similar to observations in the cortex, none of the iACS schemes demonstrated a significant impact on glial cells in the hippocampus.

*In the thalamus*, which is located deeper in brain and distant from the iACS electrodes, the induction in the neuron population was observed following ips‐iACS and bi‐iACS, as revealed by snRNA‐seq analysis. Specifically, ips‐iACS led to a 33.4% increase in neuron numbers, and bi‐iACS resulted in a 31.03% increase. However, con‐iACS did not demonstrate a significant effect, as there was only a negligible increase of −0.42% in neuron proportion when compared to sham (Figure 2M). As for other types of cells in the thalamus, the ips‐iACS and bi‐iACS showed a non‐significant trend in decreasing astrocytes, microglia, and oligodendrocyte numbers (Figure 2M).

The real‐time PCR analysis validated consistent patterns in *NeuN* expression within the thalamus after iACS treatments. In comparison to sham, there was a 46.6% increase in *NeuN* expression with ips‐iACS (*p* = 0.0430, *n* = 3 rats for each group). Despite a 38.4% increase with bi‐iACS (*p* = 0.0155, *n* = 3), this change was not statistically significant. With con‐iACS, *NeuN* expression was reduced by 0.042% (*p* = 0.9847), a result that was not statistically significant and mirrored the trends observed in the snRNA‐seq profiling (Figure 2N).

The immunofluorescence showed an increase of NeuN+ neuron by the ips‐iACS (44.56%, *p* = 0.0095, *n* = 3 rats for each group) treatment versus to sham (30.01%), but no significance was detected with either bi‐iACS (35%, *p* = 0.4187, *n* = 3) or con‐iACS (26.61%, *p* = 0.6788, *n* = 3) (Figure 3I,J). Although in a decreasing trend by ips‐iACS and bi‐iACS, there was no significant change on GFAP+ glial cells was detected by immunofluorescence across the three iACS groups (Figure 3K,L).

While ips‐iACS was still recognized as the most effective scheme for enhancing neuronal numbers, the results from snRNA‐seq, real‐time PCR, and immunofluorescence analyses indicated a weaker induction effect on neurons in the thalamus compared to that observed in the cortex and hippocampus. Additionally, the three iACS treatments demonstrated irregular effects on other cell types in the thalamus, irrespective of ips‐iACS, bi‐iACS, or con‐iACS.

Combining the results, it demonstrated that neurons were the primary cell type responding to iACS across all the three brain regions: cortex, hippocampus, and thalamus. Among the iACS schemes, ips‐iACS, which generated an EF above 150 mV/mm (in the cortex: 237.3 ± 31.6 V/m, in DG of the hippocampus: 204.9 ± 21.8 V/m, in SVZ of the thalamus: 180.7 ± 8.9 V/m). Figure 1D–G is identified as the most effective approach to increase neurons in these brain regions.

### 2.4 Rgs9 in neurons was negatively regulated by iACS

When neurons were identified as the most sensitive cell type to ips‐iACS and other schemes of iACS, we next sub‐clustered the neurons (Figures S4, S6, and S8) and compared the gene expression levels for the neurons with the ips‐iACS versus sham, separately in the cortex, hippocampus, and thalamus, to identify the differentially expressed genes under iACS.

**FIGURE 4:**
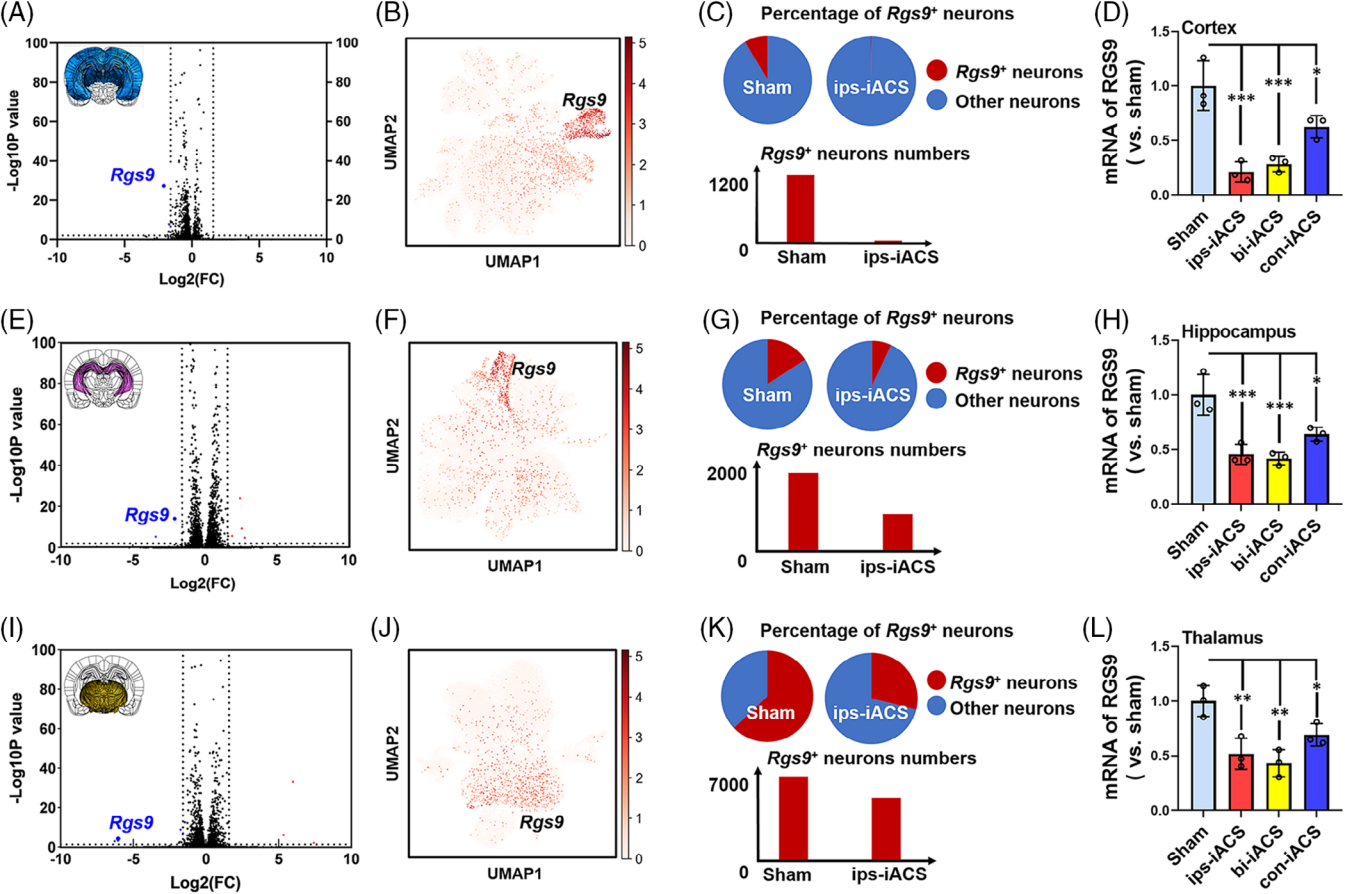
*Rgs9* gene expression difference by the intracranial alternating current stimulation (iACS) treatments. Volcano plot of gene expression changes of neurons between sham and ips‐iACS groups in the cortex (A), hippocampus (E), and thalamus (I). Significant genes were called via DESeq2 (*p* < 0.05 and change >2‐fold). Uniform manifold approximation and projection (UMAP) showed *Rgs9* expression in the cortex (B), hippocampus (F), and thalamus (J). Distribution and cell numbers of identified *Rgs9* ^+^ neurons for sham and ips‐iACS groups in the cortex (C), hippocampus (G), and thalamus (K). The mRNA expression of *Rgs9* in the iACS‐treated cortex (D), hippocampus (H), and thalamus (L) by real‐time PCR. The mRNA expression of *Rgs9* by real‐time PCR were shown as mean ± standard deviation (SD), ^***^ *p* < 0.001, ^**^ *p* < 0.01, and ^***^ *p* < 0.05 were considered as significantly different between each iACS and sham.

Here, we profiled eight sub‐clusters of neurons from the cortex (Figure S4B) and hippocampus (Figure S6B), and seven sub‐clusters of neurons from the thalamus (Figure S8B). Upon sub‐clustering analysis, we observed a distinct sub‐cluster that exhibited differential distribution between the ips‐iACS and sham groups across all three brain regions. In all three brain regions, a distinct sub‐cluster was identified, characterized by a notable change in *Rgs9* gene in neurons, following the ips‐iACS treatment (Figures 4A,B and S4A,B for the cortex, Figures 4E,F and S6A,B for the hippocampus, and Figures 4I,J and S8A,B for the thalamus). And across all the three brain regions, the *Rgs9*+ neuron populations proportions were quantified significantly decreased by ips‐iACS (Figures 4C and S4C for the cortex, Figures 4G and S6C for the hippocampus, and Figures 4K and S8C for the thalamus). Specifically, according to the snRNA‐seq, the most significant *Rgs9*+ neuron number change by ips‐iACS was quantified in the cortex, with 97.5% decrease (Figure 4C). While the other two regions demonstrated 59.8% (the hippocampus, Figure 4G) and 54.0% (the thalamus, Figure 4K) decrease of *Rgs9*+ neuron numbers.

The real‐time PCR results consistently showed a decrease in *Rgs9* expression across the cortex, hippocampus, and thalamus following iACS treatments. In the cortex, expression of *Rgs9* was reduced to 21.26% with ips‐iACS, 28.39% with bi‐iACS, and 62.53% with con‐iACS, compared to sham (100%), as shown in Figure 4D. In the hippocampus, the expression was downregulated to 45.72% with ips‐iACS, 41.59% with bi‐iACS, and 64.01% with con‐iACS, as shown in Figure 4H. Similarly, in the thalamus, *Rgs9* levels were decreased to 51.85% with ips‐iACS, 43.2% with bi‐iACS, and 69.19% with con‐iACS, as shown in Figure 4L.

**FIGURE 5:**
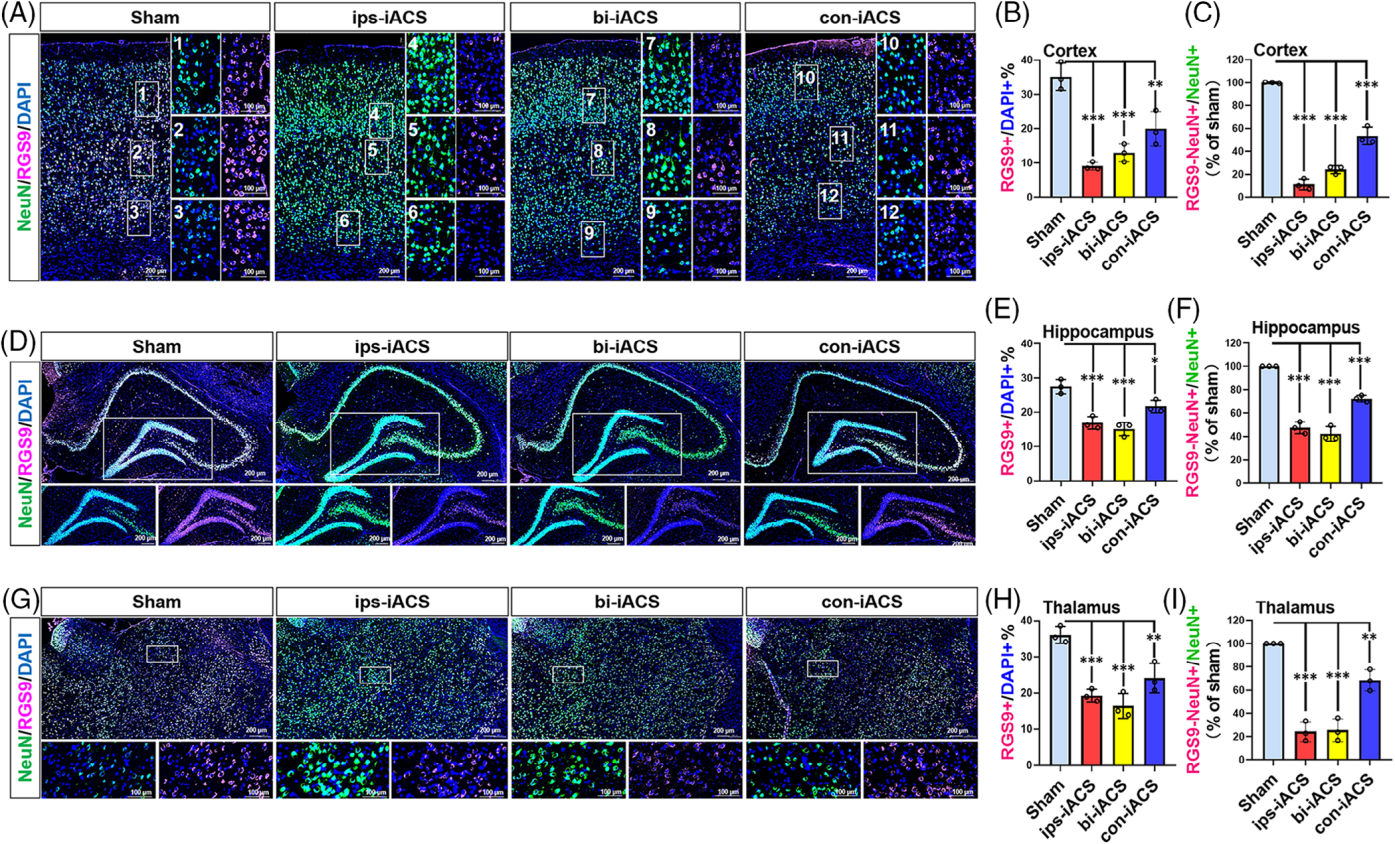
The immunofluorescence of *Rgs9*+ neurons in intracranial alternating current stimulation (iACS)‐treated rat brain. The NeuN+/RGS9 cells in the cortex (A), hippocampus (D), and thalamus (G). Quantification of RGS9+ cell count percentage in the cortex (B), hippocampus (E), and thalamus (H). Quantification of RGS9‐NeuN+/NeuN+ cell count percentage in the cortex (C), hippocampus (F), and thalamus (I). The nuclei were labeled with DAPI staining. The percentages of RGS9+/DAPI+, RGS9‐NeuN+/NeuN+ were shown as mean ± standard deviation (SD), ^***^ *p* < 0.001, ^**^ *p* < 0.01, and ^*^ *p* < 0.05 were considered as significantly different between each of the three iACS groups and sham. *n* = 3 rats for each group. Score bars: 200 or 100 µm as shown in each image.

The attenuations of *Rgs9*+ neuron proportion were further confirmed with immunofluorescence, targeting at NeuN and RGS9 (the coded protein by *Rgs9*) (Figure 5). Accordingly, the iACS treatments led to an increase of NeuN+ cells, aligning with the findings presented in Figure 3. Furthermore, there were significant decreases in both the proportions of RGS9+ cells (as measured by the ratio of RGS9+ cells to DAPI+ cells) and RGS9+ neurons (as measured by the ratio of RGS9‐NeuN co‐expressing cells to NeuN+ cells), particularly notable with ips‐iACS and bi‐iACS. Specifically in the cortex (Figure 5A), the proportion of RGS9+ cells was attenuated from 35.14% (sham) to 9.1% (ips‐iACS), 12.92% (bi‐iACS), and 19.9% (con‐iACS) (Figure 5B). And the proportion of RGS9+ neurons was also sharply decreased to 11.18% by the ips‐iACS treatment, to 24.1% by bi‐iACS, and to 53.57% by con‐iACS, compared to the sham (100%) (Figure 5C). The zoomed‐in images showed the decreasing patterns of RGS9+ neurons by the iACS treatments in different layers of the cortex (Figure 5A1–12). As for in the hippocampus (Figure 5D), the proportion of RGS9+ cells decreased from 27.5% (sham) to 16.9% (ips‐iACS), 15.11% (bi‐iACS), and 21.72% (con‐iACS) (Figure 5E). And the proportion of RGS9+ neurons was also sharply decreased to 47.49% by the ips‐iACS treatment, 42.27% by bi‐iACS, and 72.46% by con‐iACS, compared to the sham (100%) (Figure 5F). The zoomed‐in images emphasized the reduction in RGS9+ neurons in ips‐iACS and bi‐iACS groups (Figure 5D). As for in the thalamus (Figure 5G), the proportion of RGS9+ cells decreased from 36.02% (sham) to 19.29% (ips‐iACS), 16.42% (bi‐iACS), and 24.14% (con‐iACS) (Figure 5H). And the proportion of RGS9+ neurons was also sharply decreased to 24.28% by the ips‐iACS treatment, 25.57% by bi‐iACS, and 68.52% by con‐iACS, compared to the sham (100%) (Figure 5I).

Taken together, when neurons were found as the primary cell type to be affected by iACS, a specific subset of neurons was detected to exhibit a significant reduction in *Rgs9* expression. The most pronounced decrease in *Rgs9* expression was observed in response to ips‐iACS. The observed decrease in *Rgs9* expression progressively diminished from the cortex, through the hippocampus, and finally to the thalamus. This trend was indicative of a correlation with the intensity of the in‐brain derived EF generated by iACS, suggesting a dependency on EF intensity.

### 2.5 Rgs9 signaling in iACS‐induced neurons

**FIGURE 6:**
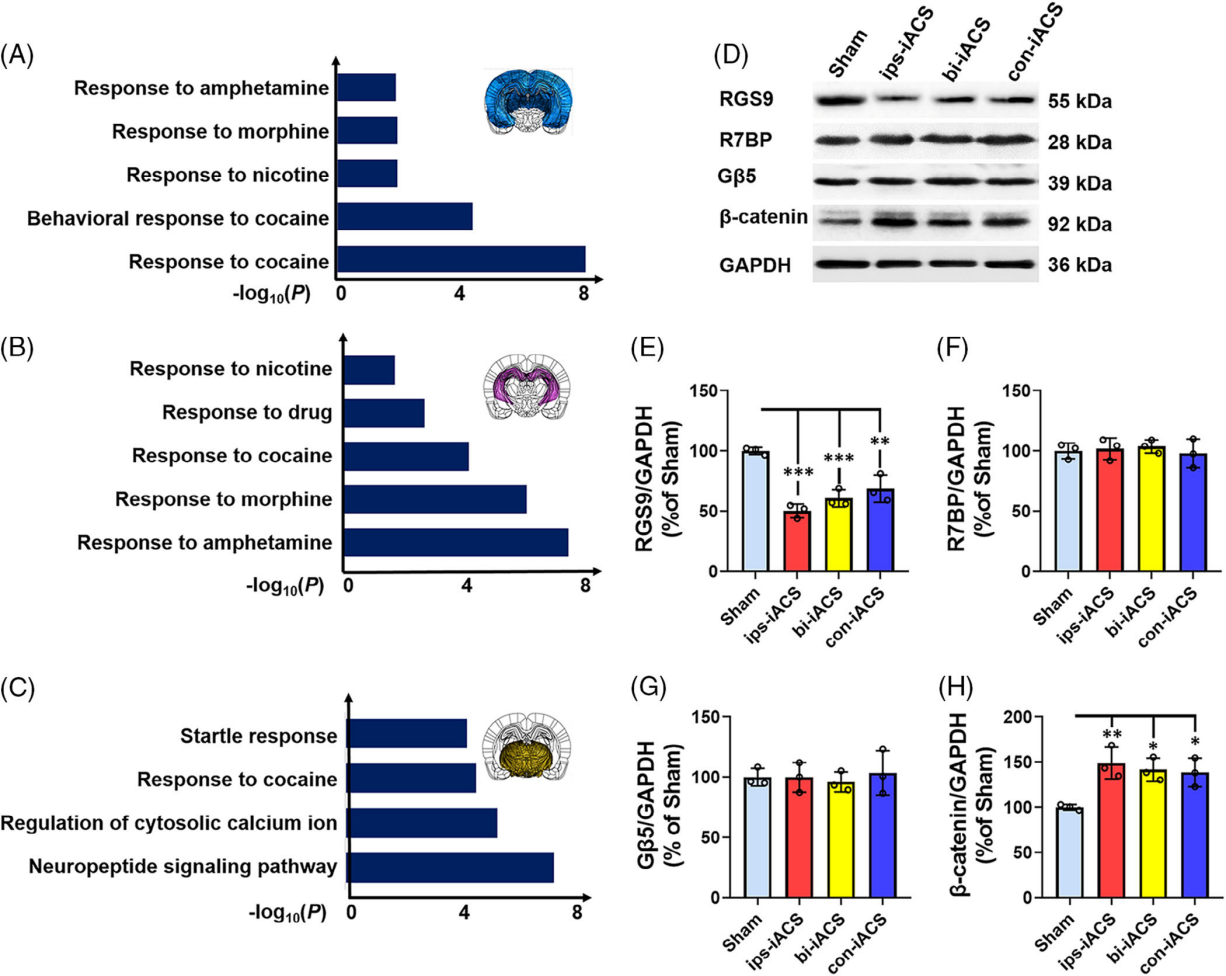
Signaling pathway analysis of intracranial alternating current stimulation (iACS) regulation on neurons. The pathway enrichment analysis of down streaming upregulated genes and pathways in the *Rgs9* ^+^ neurons from the cortex (A), hippocampus (B), and thalamus (C), using Gene Ontology (GO) biological process terms. (D) The protein expressions of RGS9, R7BP, Gβ5, and β‐catenin in the rat cortex with iACS treatments. (E) Quantification of RGS9 expression in (D). (F) Quantification of R7BP expression in (D). (G) Quantification of Gβ5 expression in (D). (H) Quantification of β‐catenin expression in (D). The protein expressions were shown as mean ± standard deviation (SD), ^***^ *p* < 0.001, ^**^ *p* < 0.01, and ^*^ *p* < 0.05 were considered as significantly different between each of the three iACS groups and sham. *n* = 3 rats for each group.

When *Rgs9* was pinpointed as a key gene responsive to iACS in neurons, we then conducted an analysis of the pathway enrichment for genes that were up regulated in *Rgs9*‐positive neurons within the cortex, hippocampus, and thalamus. Utilizing Gene Ontology (GO) analysis in conjunction with snRNA‐seq data, we found that *Rgs9* was associated with neuronal differentiation, maturation, and drug responsive processes to substances such as amphetamine, morphine, and nicotine, as shown in Figure 6A–C. Further intracellular signaling associated with iACS downregulated *Rgs9* was further examined by analyzing protein expression in the cortex tissue samples, which showed the most notable reduction in *Rgs9* following the ips‐iACS (Figure 6D–H). Along with the downregulation of *Rgs9* on the RNA level, the protein expression of RGS9 was also detected to be downregulated with the same significance (Figure 6D,E). However, the protein expressions of regulator of G protein signaling 7 binding protein (R7BP) (Figure 6D,F) and G protein subunit beta 5 (Gβ5) (Figure 6D,G) were detected with no change by any of the iACS treatments. The two proteins of R7BP and Gβ5 were recognized as the other two members of the RGS9/R7BP/Gβ5 complex, playing primary regulating roles in neuronal response to drugs and neuronal differentiation.^25^, ^26^ While, the expression of β‐catenin in nucleus, previously identified as a downstream targeting signal negatively affected by RGS9^27^ and as a regulator of neuronal differentiation in response to electrical stimulation,^28^ were found to be increased with ips‐iACS, bi‐iACS, and con‐iACS, as shown in Figure 6D,H. The results indicated that iACS could negatively regulate *Rgs9* expression, which in turn affected the functioning of the RGS9/R7BP/Gβ5 complex. However, iACS did not directly effect on either R7BP or Gβ5 components of the complex; on the other hand, iACS triggered the β‐catenin activation through downregulating *Rgs9*. Both signaling offered another potential explanation for neuronal enrichment in the iACS‐treated brain.

## 3 DISCUSSION

The EBS has been evidenced immediately safe and effective for serials of brain disorders, ever since deep brain stimulation (DBS) was approved by the Food and Drug Administration for treatment of PD, tremors, etc., in 1997.^29^ Since then, EBS have been widely used through either invasive or non‐invasive way, for neurological and neuropathological modulations. The underlying mechanisms were explained as the modulation of neuronal polarization and spiking activity by direct current stimulation (DCS),^30^ as well as the entrainment and synchronization of neuronal oscillations by ACS.^31^, ^32^ Although of the accumulating studies on neural modulating effects of DBS, DCS, and ACS, there is rarely an investigation on the comparison between global and local stimulations, the cell‐type response, and the gene expression changes under EBS. Here, we provided the first snRNA‐seq profile, separately of the rat cortex, hippocampus, and thalamus regions under either bilateral or unilateral iACS at 40 Hz. Our primary goal for this study was to identify potential cellular targets and assess the safety of these therapeutic interventions, particularly in the context of healthy cells within brains. This approach was anticipated to yield vital information that could significantly influence the treatment strategies for EBS for various neurological disorders.

Across all the cell‐type clusters with iACS, we surprisingly found neurons as the only cluster to be significantly increased, in regions of the cortex, hippocampus, and thalamus, especially with ips‐iACS. The real‐time PCR and immunofluorescence analyses corroborated this increase observed from snRNA‐seq. The other cell types, including astrocytes, oligodendrocytes, and microglia, on the other hand, were profiled either non‐influenced or irregularly changed with no significance by any scheme of iACS. These results suggested that the neurons were the major cell type to response to EBS. Furthermore, ips‐iACS was identified as the most effective stimulation for the neuronal boost, especially in the cortex. Combined with the cell‐type cluster profile and the in‐brain EF magnitude simulation and measurement data, it suggested that the unilateral iACS generating EF at 40 Hz and 100−250 V/m in the brain regions of cortex, hippocampus, and thalamus would be the most effective way to boost neurons in brain, which was consistent with our previous founds that the effect of electrical stimulation at 100 V/m to promote the neuronal differentiation of neural stem cells,^28^ and the effect of iACS stimulation at 40 Hz to boost the neurogenesis in AD mouse brain.^21^

When neurons across the cortex, hippocampus, and thalamus were identified as the primary cell type increased by iACS, it was presumed that the increased neurons by iACS were neurons or precursor cells newly formed, indicating iACS‐induced neurogenesis. The notion of neurogenesis in the adult brain, particularly in areas like the SVZ and hippocampus, has been debated but is increasingly supported by recent research. Endogenous neurogenesis can be induced by exogenous chemical and physical stimulations. Our work contributed to this discussion, showing that electric stimulation can promote neuronal differentiation from neural stem cells, and enhance endogenous neurogenesis in healthy and AD rat brain.^21^, ^22^, ^28^ For the underlying molecular mechanism, we have observed the phosphoinositide‐3‐kinase (PI3K)/Akt (also called protein kinase B or PKB)/Glycogen xynthase kinase‐3 beta (GSK‐3β)/β‐catenin pathway in previous study. Here, to further explore the underlying mechanism and primary regulating gene, we dissected the snRNA‐seq data for the gene expression changes by iACS, finding *Rgs9* as a unique downregulating gene under the ips‐iACS. *Rgs9*, also known as the regulator of G protein signaling 9, encodes the protein RGS9, a member of the RGS family of GTPase activating proteins that regulate various intracellular signaling pathways through G protein deactivation. The expression and distribution pattern suggests an enriched gene expression of *Rgs9* in brain and retina,^33^ regulating dopamine, opioid, and protein kinase A (PKA) signalings.^34^ As a member of all RGS‐related GTPase‐accelerating proteins, *Rgs9* is involved in regulation of physiological processes, including the membrane channel activity and ion flux regulations. For instance, *Rgs9* is reported to increase the intracellular calcium out‐flux by dopamine activation of the D2 receptors.^35^, ^36^ In other studies, the encoded protein RGS9 is reported to play roles in neuronal differentiation and maturation through complex with R7BP and Gβ5.^25^, ^26^ Furthermore, when β‐catenin signaling pathway controls numerous cellular processes, including neuronal differentiation and neurogenesis, RGS9 is reported to negatively regulate β‐catenin activation.^27^ Here, our results demonstrated the GO enrichment of *Rgs9* correlated to the neuronal response to amphetamine, morphine, nicotine, as well as to neuronal processes regulation, which was modulated by iACS through *Rgs9*. As there was no direct evidence linking the function of *Rgs9* and induced neurogenesis, our GO enrichment and protein expression results indicated a potential mechanism of iACS‐induced neuronal genesis through negative regulation on *Rgs9*. Further results provided new evidence to link the RGS9 with β‐catenin activation controlled neuronal differentiation, which addressed the hypothesis of iACS‐induced neurogenesis, as well as expanded the undergoing mechanism of PI3K/Akt/GSK‐3β/β‐catenin pathway from our previous study with *Rgs9* as an initial up‐streaming responsorial signaling to iACS.

In conclusion, we provided the snRNA‐seq profiles of the rat cortex, hippocampus, and thalamus under iACS at 40 Hz. The neurons were identified as the specific cell type sensitive to iACS, responding as the increased absolute neuron number and relative proportion. Interestingly, the iACS induced less than 0.1% changes on gene expression level in neurons. While *Rgs9* was identified as a unique decrease expression gene in neuron. The unilateral iACS produced a more focused local effect on *Rgs9*+ neuron proportion attenuations on the ipsilateral hemisphere. As *Rgs9* is a negative regulator for the G proteins and β‐catenin activities, our result provided a novel mechanism that iACS would be a potential treatment for enhancing the neurogenesis and neuronal sensitization, through its unique downregulation effect on *Rgs9* in neurons. This study enhanced our understanding of the effects of iACS and tACS neural modulation on healthy brain cells and genes, providing novel information on targeting cells, responding genes and efficient stimulating strategy for further applications of EBS on many brain diseases.

## 4 MATERIALS AND METHODS

### 4.1 Animal and grouping

The male Sprague–Dawley rats used for this study, at the age of 4 months, weighing at 250–300 g were housed with ad libitum access to food and water in a room maintained at a constant temperature (20°C–22°C) on a 12–12 h light–dark cycle. The animal procedures were approved by the Institutional Animal Care and Use Committee (IACUC) at Shenzhen Institutes of Advanced Technology. All efforts were made to ensure animal comfort and to reduce the number of animals used. For sham and iACS procedures, the rats were randomly divided into four groups: sham, ips‐iACS, bi‐iACS, and con‐iACS.

### 4.2 Finite element method

The FEM was used to estimate the distribution of EF in a 3D rat brain model. A simplified brain model was built based on MRI images of 118 brain structures with T2 module (available at: https://www.nitrc.org/projects/whs‐sd‐atlas). The Sim4Life platform (v7.01.8169, Zurich MedTech AG) was used to perform a quasi‐electrostatic FEM simulation to calculate the electric current distribution in the brain model.

### 4.3 Electrode placement

The electrode implanting surgery was performed on the rats from each group 24 h before the iACS session. As described previously,^21^, ^37^, ^38^ the rats received the anesthesia with 2% (v/v) isoflurane in O_2_ flow (0.2–0.3 L/min) before surgery.

Two stainless steel screws (0‐80, D.I.A., 0.067 in.) were sterilized and implanted at the coordinates—anteroposterior (AP): −4.2, mediolateral (ML): ±4.5 (for bilateral iACS) or at AP: −4.2, ML: −4.5 and AP: 1, ML: −2.5 (for unilateral iACS) (unit: mm). The coordinates were set according to “The rat brain in stereotaxic coordinates—sixth edition.”^39^ During the entire surgery, the rats were placed on a thermostatically controlled warming pad and body temperature monitored with a rectal thermometer. Depth of anesthesia was monitored every 5 min by a toe pinch to elicit a foot withdraw. For the analgesic regimen, the rats received subcutaneous Carprofen at 5 mg/kg at the time of surgery. The rats’ neurological function was assessed twice daily in the following 2 days after the surgery, and Carprofen was administered if rats showed signs of pain or stress.

### 4.4 Intracranial AC stimulation

The iACS was delivered through the screw electrodes 24‐h post the implantation surgery. For the treatment, the rats were anesthetized with 2% (v/v) isoflurane in O^2^ flow (0.2–0.3 L/min). The iACS was performed with the parameters: 40 Hz at 250 µA (signal produced and monitored by the Neuroelectrics Starstim), for 1 h per day, for 7 days. The sham rats were processed with 40 Hz at 250 µA for 59 s per day, for 7 days, as the fake stimulation control.

### 4.5 Measurement of in‐brain derived EF

For EF measurement, the rat was separately anesthetized with 2% (v/v) isoflurane in O^2^ flow (0.2–0.3 L/min) and placed in the stereotaxic apparatus. Two stainless screw electrodes were sterilized and implanted into skull as the stimulating electrodes. Three pairs of Ag/AgCl measuring electrodes implantation according to “The rat brain in stereotaxic coordinates—6th edition.”^37^, ^39^ In brief, for the con‐iACS group, the coordinates of the three pairs of Ag/AgCl measuring electrodes were set at AP: −3, ML: 2.3, DV: 1.5 and AP: −2, ML: 2.3, dorsoventral (DV): 1.5; AP: −3, ML: 1.5, DV: 3.8 and AP: −4, ML: 1.5, DV: 3.8; AP: −0.5, ML: 1.8, DV: 4.5 and AP: 0.5, ML: 1.5, DV: 4.5. For the ips‐iACS group, AP: −3, ML: −2.3, DV: 1.5 and AP: −2, ML: −2.3, DV: 1.5; AP: −3, ML: −1.5, DV: 3.8 and AP: −4, ML: −1.5, DV: 3.8; AP: −0.5, ML: −1.8, DV: 4.5 and AP: 0.5, ML: −1.5, DV: 4.5. For the bi‐iACS group, AP: −3, ML: −2.3, DV: 1.5 and AP: −2, ML: −2.3, DV: 1.5; AP: −3, ML: −1.5, DV: 3.8 and AP: −4, ML: −1.5, DV: 3.8; AP: −0.5, ML: −1.8, DV: 4.5 and AP: 0.5, ML: −1.5, DV: 4.5 (unit: mm). For measurement, the iACS at 40 Hz, 25−500 µA was delivered through the paired stimulating electrodes. And the three pairs of measuring electrodes will separately connect to an oscilloscope to record the produced EF in deep brain regions of the forebrain cortex, hippocampus, and SVZ. The measurements were analyzed and compared with the computer simulated EF at the same regions.

### 4.6 Brain tissue collection

Following the completion of a 7‐day iACS stimulation trial, brain region samples from a consistent batch of experimental rats were collected for snRNA‐seq profiling. Specifically, this encompassed 24 samples, corresponding to two rats per group across four experimental groups, and three distinct brain regions (Table S1). For brain collection, the CO_2_ overdose euthanized rats received cardiac perfusion and fixation with iced DPBS. The brain tissues were dissected on ice bath for the cortex, hippocampus, and thalamus collection. The three regions of each rat brain were separately used for the following snRNA‐seq analysis. The tissues were rinsed with pre‐cooled PBSE, a phosphate‐buffered saline (PBS) buffer containing 2 mM egtazic acid (EGTA), before commencing the experiment. Nucleus isolation was performed using GEXSCOPE Nucleus Separation Solution (Singleron Biotechnologies) following the manufacturer's product manual. Here is the summary: take 100 mg of frozen tissue, wash with 1 mL pre‐cooled PBSE to remove impurities, and repeat. Add 50 µL pre‐cooled nuclear extraction buffer, cut tissue with surgical scissors, and incubate on ice for 1−2 min. Add 950 µL pre‐chilled nuclear extraction buffer, incubate on ice for 5 min, mix two to three times. Filter into a 50 mL tube, rinse the filter with 9 mL pre‐cooled nuclear washing solution PBSE. Centrifuge at 200 rcf at 4°C for 2 min and transfer 9 mL supernatant to a new tube. Centrifuge at 500 rcf at 4°C for 5 min, leave supernatant, and mix pellet with 50 µL pre‐chilled PBS. Add 200 µL DAPI staining solution, mix, and react for 2 min on ice in the dark. Add 5 mL pre‐cooled PBSE, filter into a new tube, centrifuge at 500 rcf at 4°C for 5 min and remove excess liquid. Add 100–200 µL pre‐cooled PBS, gently resuspend nucleus pellet. Adjust nucleus concentration to 3–4 × 10^5^ nuclei/mL with PBS (added with RNase inhibitor and DL‐dithiothereitol (DTT)), ready for use. The isolated nuclei were then resuspended in PBSE at a concentration of 10^6^ nuclei per 400 µL, filtered through a 40 µm cell strainer, and counted using Trypan blue. DAPI staining (1:1000; Thermo Fisher Scientific, D1306) was performed on the PBSE‐enriched nuclei, with nuclei being identified as DAPI‐positive singlets. The single‐nucleus suspension concentration was adjusted to 3–4 × 10^5^ nuclei/mL in PBS and loaded onto a microfluidic chip (GEXSCOPE Single Nucleus RNA‐seq Kit, Singleron Biotechnologies). The resulting snRNA‐seq libraries were prepared in accordance with the manufacturer's instructions (Singleron Biotechnologies) and sequenced on an Illumina HiSeq X10 instrument to a sequencing depth of at least 50,000 reads per cell, using 150‐bp paired‐end (PE150) reads.

### 4.7 snRNA‐seq data analysis

The gene expression matrices were generated from raw reads using scopetools (https://anaconda.org/singleronbio/scopetools). The first step involved filtering out reads without polyT tails and extracting cell barcodes and unique molecular identifiers (UMIs). Adapters and polyA tails were trimmed before aligning the reads to the pre‐mRNA reference (Ensemble, Rnor6.0 genome). Then reads with the same cell barcode, UMI, and gene were grouped together to count the number of UMIs per gene per cell. The cell number was determined using the “knee” method, a standard quality control approach for snRNA‐seq, which identifies the inflection point (or “knee”) on a plot of the number of UMIs versus the number of cells. Barcodes to the left of the knee point, indicating high‐quality cells, were retained for further analysis, while those to the right were excluded. The cell barcode files from the filtered matrix, corresponding to the cell fraction, were then analyzed using Scanpy v1.9.^40^

### 4.8 Real‐time PCR

Total RNA of brain tissue was extracted from the treated rats or cDNA synthesis using PrimeScript RT Master Mix (Perfect Real Time) (TAKARA RR036A).^28^ Real‐time PCR reactions were performed using the Roche LightCycler96 and SYBR Green Premix Pro Taq HS qPCR (ACCURATE BIOLOGY AG11701) Kit. The primer sequence can be found in Table 1. The cycling condition were set as: an initial preincubation step at 95°C for 2 min, followed by 40 cycles of two‐step amplification at 95°C for 15 s, and melting stage can be divided into three parts, 95°C for 15 s, 60°C for 15 s, 95°C for 15 s, and finally a cooling stage of 37°C for 30 s. The mRNA expression of GAPDH was used as an internal control and the expression levels of target genes were normalized to a control sample using the 2^−ΔΔct^ method for relative quantification of gene expression.

**TABLE 1:** List of the primers used in this study.

Primers		Sequence 5′−3′
NeuN	Forward	CAGTACCCTCCTCCACCTCA
	Reverse	TGCTGCTTCATCTGCCTGC
Rgs9	Forward	ATCATGTCTGGCTGCCTTCC
	Reverse	GTTGGGACCTCCACCAACTT
GAPDH	Forward	TGATTCTACCCACGGCAAGTT
	Reverse	TGATGGGTTTCCCATTGATGA

### 4.9 Immunofluorescence

As described previously,^21^ the brain slices were fixed in 4% paraformaldehyde (PFA) for 30 min, and then permeabilized within 0.1% Triton X‐100 (Sigma–Aldrich) for another 30 min. After blocking the non‐specific proteins with 3% bovine serum albumin (BSA)–PBS at room temperature for 1 h, the slices were incubated with the primary antibodies: NeuN (1:200, #24307S, Cell Signaling Technology), GFAP (1:200, #3670, Cell Signaling Technology), RGS9 (1:200, sc‐377252, Santa Cruz Biotech) at 4°C, overnight. The slices were then transferred into the 3% BSA–PBS solution containing goat secondary antibody (1:200, Alex Fluor 594/488, #A‐32731, #A‐11032, Invitrogen, Alexa Fluor 647, ab150123, Abcam). DAPI was applied to label the nucleus. The fluorescence was detected with a NIKON ECLIPSE C1 microscope and scanned by the 3DHISTECH Digital Scanner (Pannoramic MIDI). Counting of cells was performed with ImageJ software, for analysis of the neuronal and glial marker genes in the cortex, hippocampus, and thalamus.

### 4.10 Western blotting

The rat brain tissue lysate was collected after treatments for Western blotting. Specifically, the lysate samples were subjected to 5%–10% SDS–PAGE gel electrophoresis. Protein bands were then transferred onto a polyvinylidene difluoride (PVDF) membrane (0.45 µm, Millipore), followed by blocking in a 5% fat‐free milk–1X Tris‐buffered saline with 0.1% Tween20 (TBST) buffer at room temperature for 1 h. The membrane was then incubated overnight at 4°C with primary antibodies targeting RGS9 (1:1000, ab108975, Abcam), β‐catenin (1:5000, 66379‐1‐1 g, Proteintech), R7BP (1:1000, ABIN2774539, antibodies‐online.com), Gβ5 (1:1000, 11045‐2‐AP, Proteintech), and GAPDH (1:4000, 10494‐1‐AP, Proteintech). Afterward, the membrane was incubated with horseradish peroxidase (HRP)‐conjugated secondary antibodies (AS003 and AS014, ABclonal) at room temperature for 1 h. GAPDH was used as a loading control. The antigen–antibody complexes were detected using an ECL reagent kit (SQ201, EpiZyme). The protein bands were scanned using Bio‐Rad ChemiDoc XRS+ Gel imaging system (Bio‐Rad). Protein analysis was performed using ImageJ software.

### 4.11 Statistics

Data analysis was performed using GraphPad Prism 8 (GraphPad Software, Inc.), which adheres to a general linear model. Alpha level for type I error was set at 0.05 for rejecting null hypotheses. Data were expressed as mean ± standard error (SD). NeuN, GFAP, and RGS9 expressions were separately analyzed by one‐way analysis of variance for each group, followed by a Tukey's honestly significant difference post hoc analysis for the sham and iACS group comparisons.

## AUTHOR CONTRIBUTIONS

Q.L. and Y.W. designed the study. Q.L., Y.W., and B.S. provided the research fundings. Q.L., Y.C.M., and Q.L.Z. conducted the animal surgery, brain stimulation, PCR, and immunofluorescence. Y.W. performed the snRNA‐seq data analysis. Q.L. and Y.W. wrote up the manuscript and did the proof reading. All the authors have read and approved the final manuscript.

## CONFLICT OF INTEREST STATEMENT

Some authors are listed inventors of Chinese Patent, application no. CN202211380393.4 to Q.L., B.S., and Y.W. The remaining authors declare they have no conflicts of interest.

## ETHICS STATEMENT

All animal experiments were approved by the Institutional Animal Care and Use Committee (IACUC) of Shenzhen Institutes of Advanced Technology (SIAT), Chinese Academy of Sciences Research Ethics Committees (approval number: SIAT‐IACUC‐210113‐YGS‐LQ‐A1510).

## CONSENT FOR PUBLICATION

All the authors agreed with this publication.

## Supporting information

Supporting Information

## Data Availability

The RNA‐seq data were deposited in GEO (GSE253672). Other data included in this study are available upon request from the corresponding authors.

## References

[1] Wischnewski M , Alekseichuk I , Opitz A . Neurocognitive, physiological, and biophysical effects of transcranial alternating current stimulation. Trends Cogn Sci. 2023;27(2):189‐205. 36543610 10.1016/j.tics.2022.11.013 PMC9852081

[2] Grossman N , Bono D , Dedic N , et al. Noninvasive deep brain stimulation via temporally interfering electric fields. Cell. 2017;169(6):1029‐1041.e1016. 28575667 10.1016/j.cell.2017.05.024 PMC5520675

[3] Paulk AC , Zelmann R , Crocker B , et al. Local and distant cortical responses to single pulse intracranial stimulation in the human brain are differentially modulated by specific stimulation parameters. Brain Stimul. 2022;15(2):491‐508. 35247646 10.1016/j.brs.2022.02.017 PMC8985164

[4] Mosilhy EA , Alshial EE , Eltaras MM , et al. Non‐invasive transcranial brain modulation for neurological disorders treatment: a narrative review. Life Sci. 2022;307:120869. 35940222 10.1016/j.lfs.2022.120869

[5] Antal A , Luber B , Brem AK , et al. Non‐invasive brain stimulation and neuroenhancement. Clin Neurophysiol Pract. 2022;7:146‐165. 35734582 10.1016/j.cnp.2022.05.002 PMC9207555

[6] Menardi A , Rossi S , Koch G , et al. Toward noninvasive brain stimulation 2.0 in Alzheimer's disease. Ageing Res Rev. 2022;75:101555. 34973457 10.1016/j.arr.2021.101555 PMC8858588

[7] Peeters J , Boogers A , Van Bogaert T , et al. Electrophysiologic evidence that directional deep brain stimulation activates distinct neural circuits in patients with Parkinson disease. Neuromodulation. 2023;26(2):403‐413. 35088733 10.1016/j.neurom.2021.11.002

[8] Liu Y , Tang C , Wei K , et al. Transcranial alternating current stimulation combined with sound stimulation improves the cognitive function of patients with Alzheimer's disease: a case report and literature review. Front Neurol. 2022;13:962684. 36212652 10.3389/fneur.2022.962684 PMC9539040

[9] Pini L , Pizzini FB , Boscolo‐Galazzo I , et al. Brain network modulation in Alzheimer's and frontotemporal dementia with transcranial electrical stimulation. Neurobiol Aging. 2022;111:24‐34. 34942516 10.1016/j.neurobiolaging.2021.11.005

[10] Dhaynaut M , Sprugnoli G , Cappon D , et al. Impact of 40 Hz transcranial alternating current stimulation on cerebral Tau burden in patients with Alzheimer's disease: a case series. J Alzheimers Dis. 2022;85(4):1667‐1676. 34958021 10.3233/JAD-215072 PMC9023125

[11] Wang LC , Wei WY , Ho PC . Short‐term cortical electrical stimulation during the acute stage of traumatic brain injury improves functional recovery. Biomedicines. 2022;10(8):1965. 36009512 10.3390/biomedicines10081965 PMC9405844

[12] Yuan K , Ti CE , Wang X , et al. Individual electric field predicts functional connectivity changes after anodal transcranial direct‐current stimulation in chronic stroke. Neurosci Res. 2023;186:21‐32. 36220454 10.1016/j.neures.2022.10.003

[13] Hsu G , Shereen AD , Cohen LG , Parra LC . Robust enhancement of motor sequence learning with 4 mA transcranial electric stimulation. Brain Stimul. 2022;16(1):56‐67. 36574814 10.1016/j.brs.2022.12.011 PMC10171179

[14] Hyldahl F , Hem‐Jensen E , Rahbek UL , Tritsaris K , Dissing S . Pulsed electric fields stimulate microglial transmitter release of VEGF, IL‐8 and GLP‐1 and activate endothelial cells through paracrine signaling. Neurochem Int. 2023;163:105469. 36592699 10.1016/j.neuint.2022.105469

[15] Martorell AJ , Paulson AL , Suk HJ , et al. Multi‐sensory gamma stimulation ameliorates Alzheimer's‐associated pathology and improves cognition. Cell. 2019;177(2):256‐271.e222. 30879788 10.1016/j.cell.2019.02.014 PMC6774262

[16] Iaccarino HF , Singer AC , Martorell AJ , et al. Gamma frequency entrainment attenuates amyloid load and modifies microglia. Nature. 2016;540(7632):230‐235. 27929004 10.1038/nature20587 PMC5656389

[17] Adaikkan C , Middleton SJ , Marco A , et al. Gamma entrainment binds higher‐order brain regions and offers neuroprotection. Neuron. 2019;102(5):929‐943.e928. 31076275 10.1016/j.neuron.2019.04.011 PMC6697125

[18] Soula M , Martin‐Avila A , Zhang Y , et al. Forty‐hertz light stimulation does not entrain native gamma oscillations in Alzheimer's disease model mice. Nat Neurosci. 2023;26(4):570‐578. 36879142 10.1038/s41593-023-01270-2 PMC10839995

[19] Nissim NR , Pham DVH , Poddar T , Blutt E , Hamilton RH . The impact of gamma transcranial alternating current stimulation (tACS) on cognitive and memory processes in patients with mild cognitive impairment or Alzheimer's disease: a literature review. Brain Stimul. 2023;16(3):748‐755. 37028756 10.1016/j.brs.2023.04.001 PMC10862495

[20] Voroslakos M , Takeuchi Y , Brinyiczki K , et al. Direct effects of transcranial electric stimulation on brain circuits in rats and humans. Nat Commun. 2018;9(1):483. 29396478 10.1038/s41467-018-02928-3 PMC5797140

[21] Liu Q , Jiao Y , Yang W , et al. Intracranial alternating current stimulation facilitates neurogenesis in a mouse model of Alzheimer's disease. Alzheimers Res Ther. 2020;12(1):89. 32703308 10.1186/s13195-020-00656-9 PMC7376967

[22] Liu Q , Contreras A , Afaq MS , et al. Intensity‐dependent gamma electrical stimulation regulates microglial activation, reduces beta‐amyloid load, and facilitates memory in a mouse model of Alzheimer's disease. Cell Biosci. 2023;13(1):138. 37507776 10.1186/s13578-023-01085-5 PMC10386209

[23] Mathys H , Davila‐Velderrain J , Peng Z , et al. Single‐cell transcriptomic analysis of Alzheimer's disease. Nature. 2019;570(7761):332‐337. 31042697 10.1038/s41586-019-1195-2 PMC6865822

[24] Armand EJ , Li J , Xie F , Luo C , Mukamel EA . Single‐cell sequencing of brain cell transcriptomes and epigenomes. Neuron. 2021;109(1):11‐26. 33412093 10.1016/j.neuron.2020.12.010 PMC7808568

[25] Anderson GR , Lujan R , Semenov A , et al. Expression and localization of RGS9‐2/G 5/R7BP complex in vivo is set by dynamic control of its constitutive degradation by cellular cysteine proteases. J Neurosci. 2007;27(51):14117‐14127. 18094251 10.1523/JNEUROSCI.3884-07.2007 PMC6673529

[26] Mitsi V , Terzi D , Purushothaman I , et al. RGS9‐2–controlled adaptations in the striatum determine the onset of action and efficacy of antidepressants in neuropathic pain states. Proc Natl Acad Sci U S A. 2015;112(36):E5088‐E5097. 26305935 10.1073/pnas.1504283112 PMC4568688

[27] Feigin ME , Malbon CC . RGS19 regulates Wnt‐beta‐catenin signaling through inactivation of Galpha(o). J Cell Sci. 2007;120(pt 19):3404‐3414. 17855383 10.1242/jcs.011254

[28] Liu Q , Telezhkin V , Jiang W , et al. Electric field stimulation boosts neuronal differentiation of neural stem cells for spinal cord injury treatment via PI3K/Akt/GSK‐3beta/beta‐catenin activation. Cell Biosci. 2023;13(1):4. 36624495 10.1186/s13578-023-00954-3 PMC9830810

[29] FDA . FDA Approves Brain Implant to Help Reduce Parkinson's Disease and Essential Tremor Symptoms . Retrieved May 23, 2016.

[30] Ukueberuwa D , Wassermann EM . Direct current brain polarization: a simple, noninvasive technique for human neuromodulation. Neuromodulation. 2010;13(3):168‐173. 21992828 10.1111/j.1525-1403.2010.00283.x PMC7266012

[31] Krause MR , Vieira PG , Thivierge JP , Pack CC . Brain stimulation competes with ongoing oscillations for control of spike timing in the primate brain. PLoS Biol. 2022;20(5):e3001650. 35613140 10.1371/journal.pbio.3001650 PMC9132296

[32] Asamoah B , Khatoun A , Mc Laughlin M . tACS motor system effects can be caused by transcutaneous stimulation of peripheral nerves. Nat Commun. 2019;10(1):266. 30655523 10.1038/s41467-018-08183-w PMC6336776

[33] Sakloth F , Polizu C , Bertherat F , Zachariou V . Regulators of G protein signaling in analgesia and addiction. Mol Pharmacol. 2020;98(6):739‐750. 32474445 10.1124/mol.119.119206 PMC7662521

[34] Bonsi P , Ponterio G , Vanni V , et al. RGS9‐2 rescues dopamine D2 receptor levels and signaling in DYT1 dystonia mouse models. EMBO Mol Med. 2019;11(1):e9283. 30552094 10.15252/emmm.201809283 PMC6328939

[35] Clark MJ , Harrison C , Zhong H , Neubig RR , Traynor JR . Endogenous RGS protein action modulates mu‐opioid signaling through Galphao. Effects on adenylyl cyclase, extracellular signal‐regulated kinases, and intracellular calcium pathways. J Biol Chem. 2003;278(11):9418‐9425. 12524446 10.1074/jbc.M208885200

[36] Cabrera‐Vera TM , Hernandez S , Earls LR , et al. RGS9‐2 modulates D2 dopamine receptor‐mediated Ca^2+^ channel inhibition in rat striatal cholinergic interneurons. Proc Natl Acad Sci U S A. 2004;101(46):16339‐16344. 15534226 10.1073/pnas.0407416101 PMC528982

[37] Feng JF , Liu J , Zhang L , et al. Electrical guidance of human stem cells in the rat brain. Stem Cell Rep. 2017;9(1):177‐189. 10.1016/j.stemcr.2017.05.035 PMC5511115 28669601

[38] Feng JF , Liu J , Zhang XZ , et al. Guided migration of neural stem cells derived from human embryonic stem cells by an electric field. Stem Cells. 2012;30(2):349‐355. 22076946 10.1002/stem.779 PMC4437764

[39] Paxinos G , Watson C , The rat brain in stereotaxic coordinates: hard cover edition. *Online via Elsevier*. 2006.

[40] Wolf FA , Angerer P , Theis FJ . SCANPY: large‐scale single‐cell gene expression data analysis. Genome Biol. 2018;19(1):15. 29409532 10.1186/s13059-017-1382-0 PMC5802054

