# Supplementary material for: Transcriptomic analysis of rat brain response to alternating current electrical stimulation: unveiling insights via single‐nucleus RNA sequencing
Source: MedComm (2020). 2024 Mar 15;5(4):e514. doi: 10.1002/mco2.514 (PMC10943177; doi:10.1002/mco2.514)
Supplement: Supplementary file 1 — Supporting Information [file MCO2-5-e514-s001.docx]

**Supplementary Materials**

**Transcriptomic analysis of rat brain response to alternating current electrical stimulation: unveiling insights via single-nucleus RNA sequencing**

**Yan Wang*, Yongchao Ma, Qiuling Zhong, Bing Song, Qian Liu***

Institute of Biomedical and Health Engineering, Shenzhen Institutes of Advanced Technology (SIAT), Chinese Academy of Sciences, Shenzhen, China

**Corresponding authors:**

Dr Qian Liu, [liuqian@siat.ac.cn](mailto:liuqian@siat.ac.cn); Dr Yan Wang, [yan.wang@siat.ac.cn](mailto:yan.wang@siat.ac.cn)

**Table S1. Rat brain samples for snRNA-seq (24 samples)**

| **Sample No.** | **Animal No.** | **Group** | **Region**  **(single hemisphere)** | **sample weight  (mg)** |
| --- | --- | --- | --- | --- |
| 1 | 1 | sham | Cortex | 108 |
| 2 |  |  | Hippocampus | 53 |
| 3 |  |  | Thalamus | 99 |
| 4 | 2 | bi-iACS | Cortex | 121 |
| 5 |  |  | Hippocampus | 63 |
| 6 |  |  | Thalamus | 95 |
| 7 | 6 | ips-iACS | Cortex | 49 |
| 8 |  |  | Hippocampus | 40 |
| 9 |  |  | Thalamus | 64 |
| 10 | 7 | con-iACS | Cortex | 75 |
| 11 |  |  | Hippocampus | 66 |
| 12 |  |  | Thalamus | 74 |
| 13 | 8 | sham | Cortex | 85 |
| 14 |  |  | Hippocampus | 50 |
| 15 |  |  | Thalamus | 85 |
| 16 | 5 | con-iACS | Cortex | 57 |
| 17 |  |  | Hippocampus | 64 |
| 18 |  |  | Thalamus | 84 |
| 19 | 9 | ips-iACS | Cortex | 109 |
| 20 |  |  | Hippocampus | 36 |
| 21 |  |  | Thalamus | 54 |
| 22 | 10 | bi-iACS | Cortex | 122 |
| 23 |  |  | Hippocampus | 72 |
| 24 |  |  | Thalamus | 75 |

**Figure S1**


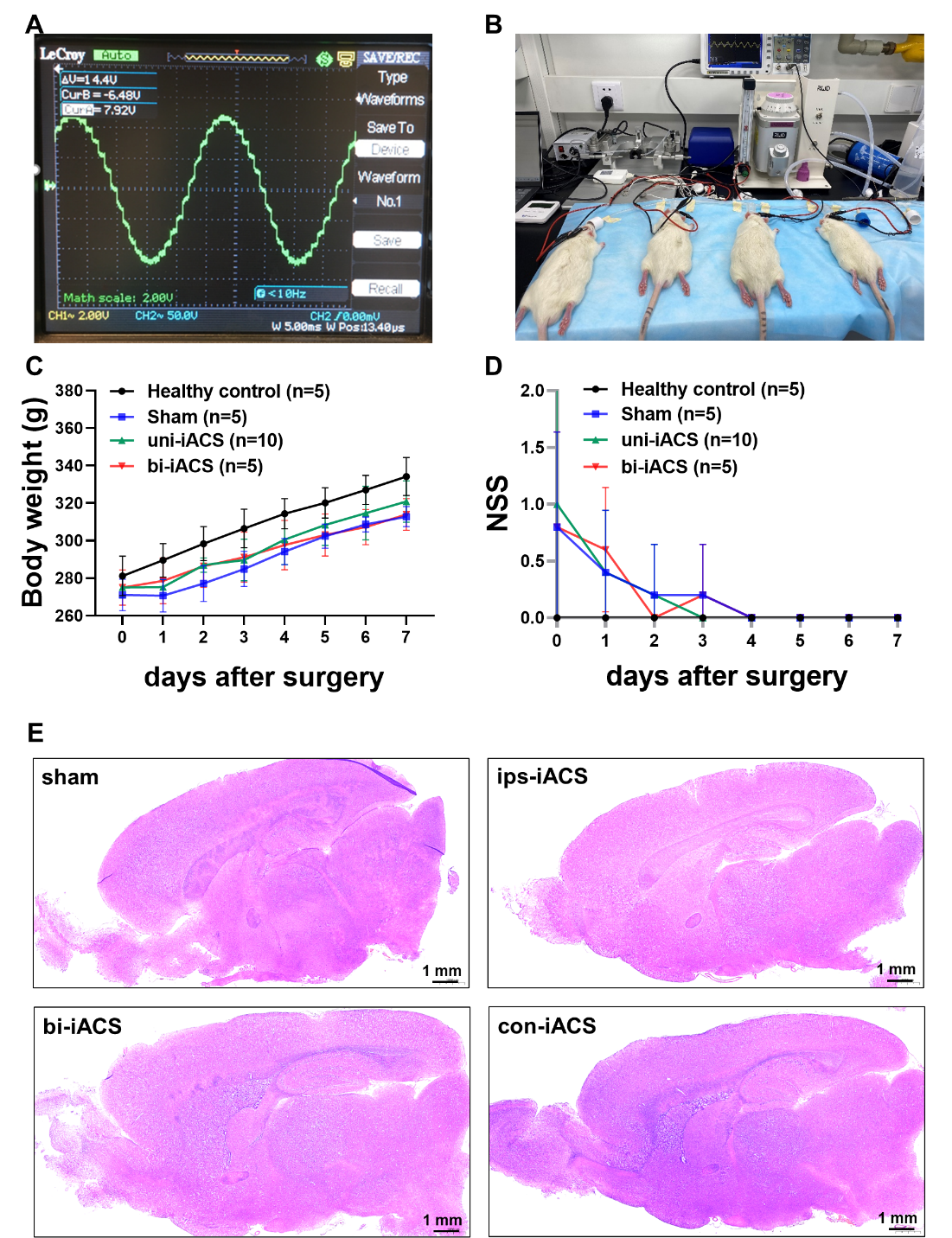


**Figure S1. The iACS for rat brain stimulation.** **A-B)** The sinusoidal alternating current was intracranially delivered to the rat brain with parameters: 40 Hz, 250 μA, 1 h per day for seven days, through the paired either bilateral or unilateral electrodes. **C)** Body weight record of the experimental rats with the electrode implantation surgery and 7-day iACS trials. **D)** The Neurological Severity Score (NSS) assessment of the rats with the electrode implantation surgery and 7-day iACS trials. n = 5 rats for each group. * P < 0.05 was considered as significantly different between the healthy/sham and iACS groups. **G)** The H&E staining of the rat brains with sham or iACS treatment. Score bars: 1 mm.

**Figure S2
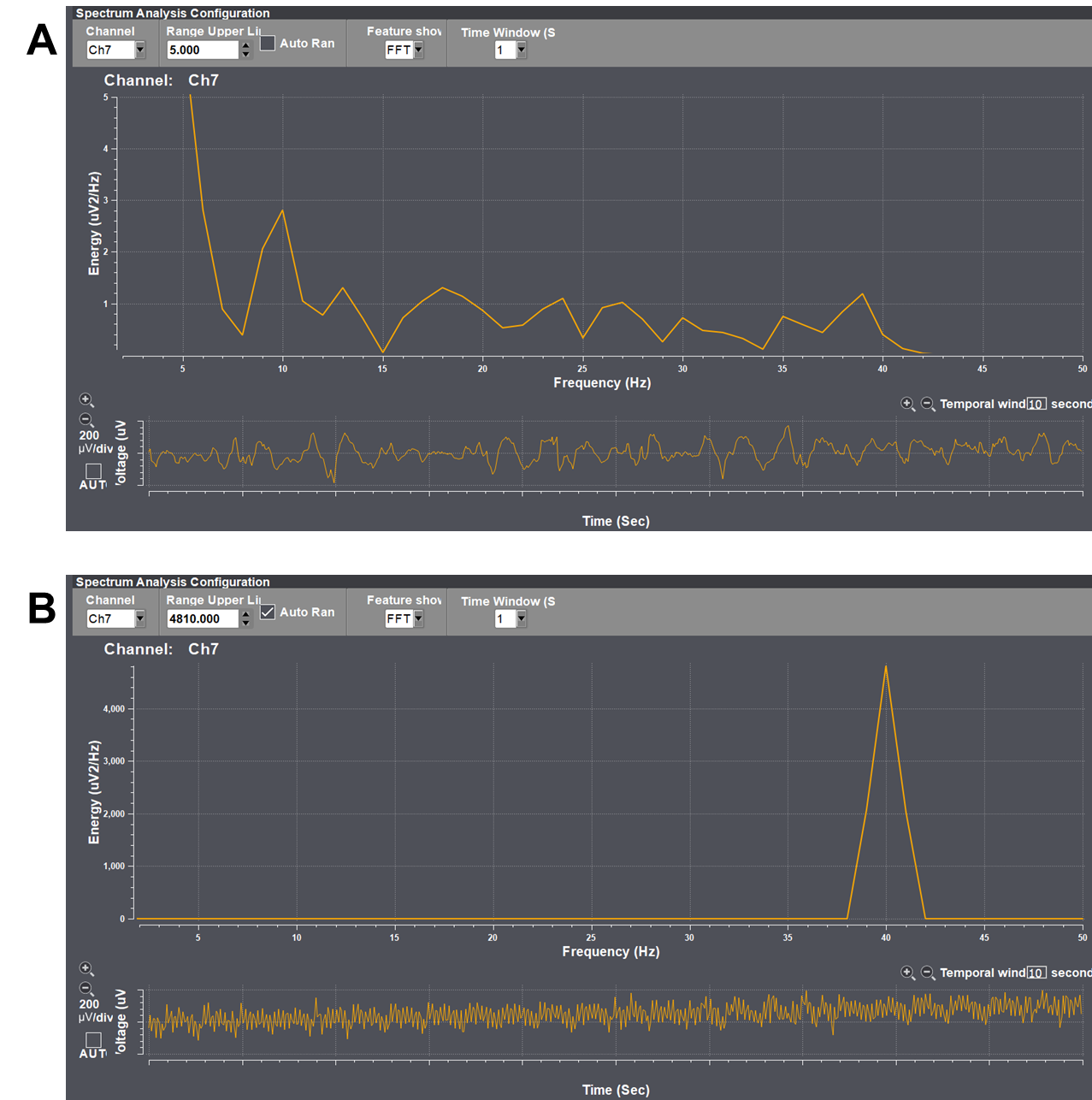
**

**Figure S2. The real-time EEG read of the iACS treating rat.** **A)** The spectrum of EEG before iACS. **B)** The spectrum of EEG during the iACS was on.

**Figure S3
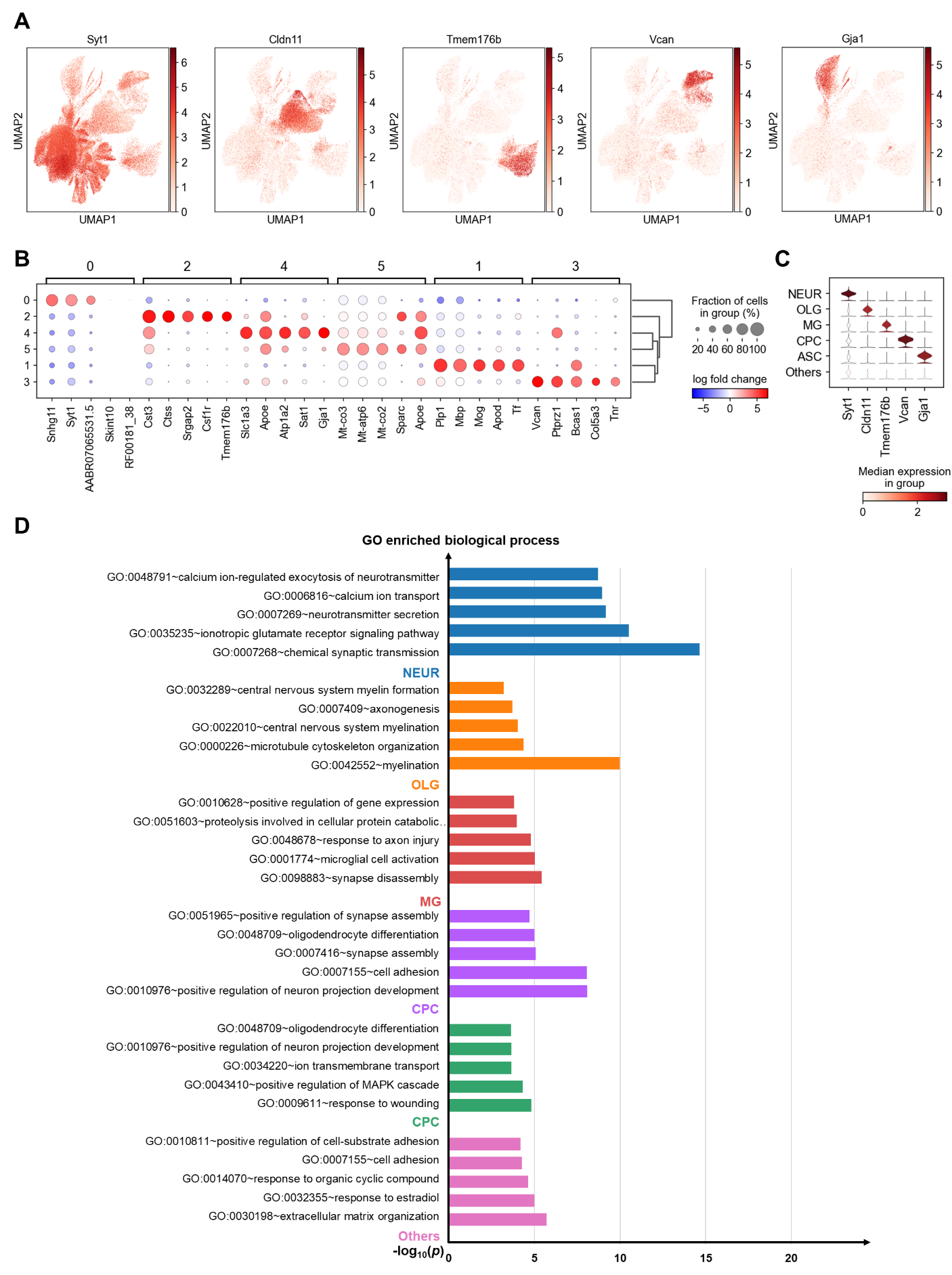
**

**Figure S3. Rats’ cortex snRNA-seq data analysis.** **A)** Marker gene expression in each cluster by UMAP analysis. **B)** Clusters are listed on y-axis, showing unbiased gene expression for the top 4 genes per cluster identified by log Fold Change; genes are listed along the x-axis. Dot size reflects percentage of cells in a cluster expressing each gene; dot color reflects expression level. **C)** Violin plots showing the representative marker genes in each cluster. **D)** Functional annotation (DAVID) of each cluster-enriched genes. P values were calculated using DAVID.

**Figure S4**

**
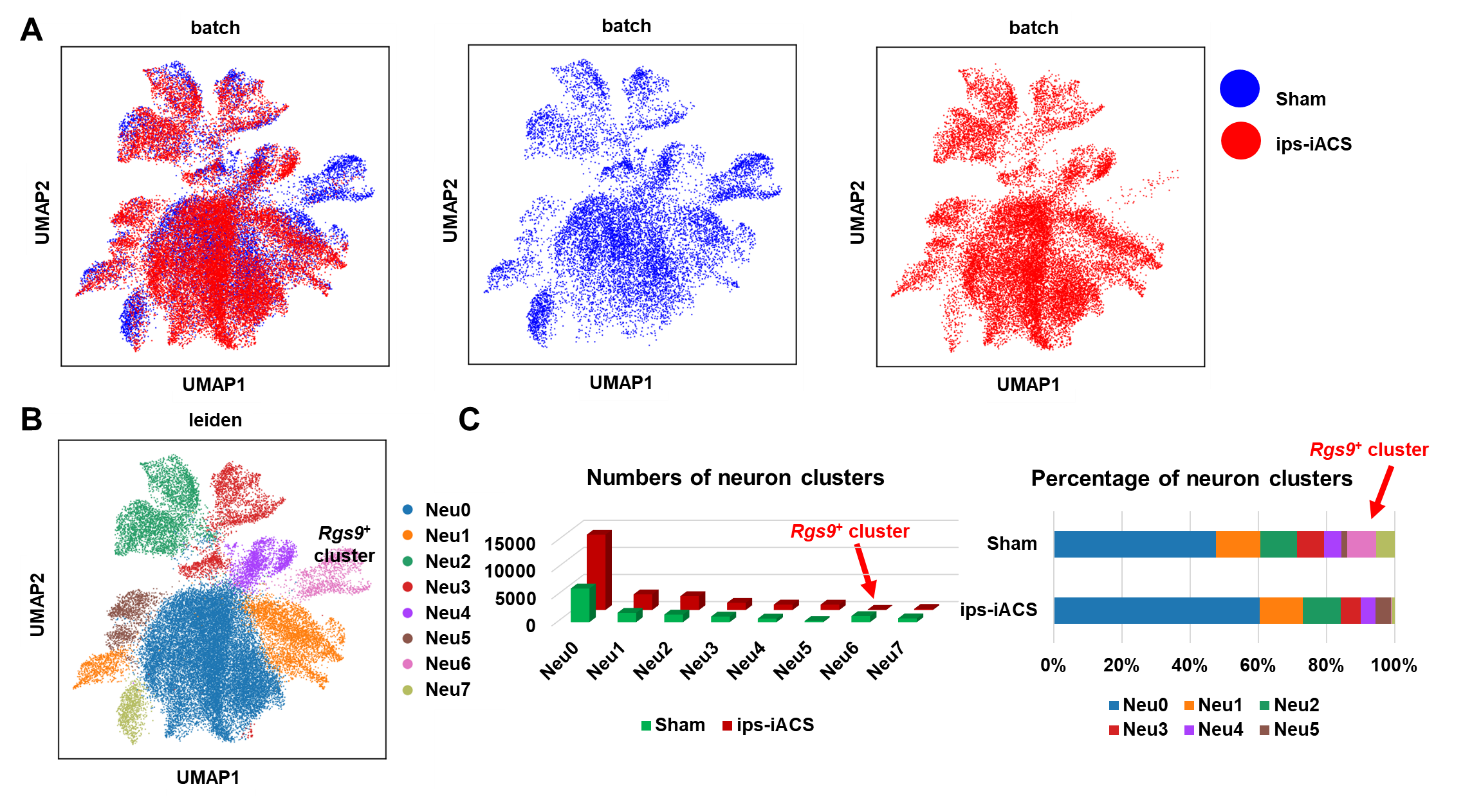
**

**Figure S4. The snRNA-seq data analysis of neurons in the cortex.** **A-C)** Uniform Manifold Approximation and Projection (UMAP) embedding of analyzed transcriptomes from sham and ips-iACS cortex neurons annotated by (**A**) treatment of distribution as well as (**B**) neuron subpopulations. (**C**) Neuron numbers and distributions of identified subpopulations.

**Figure S5
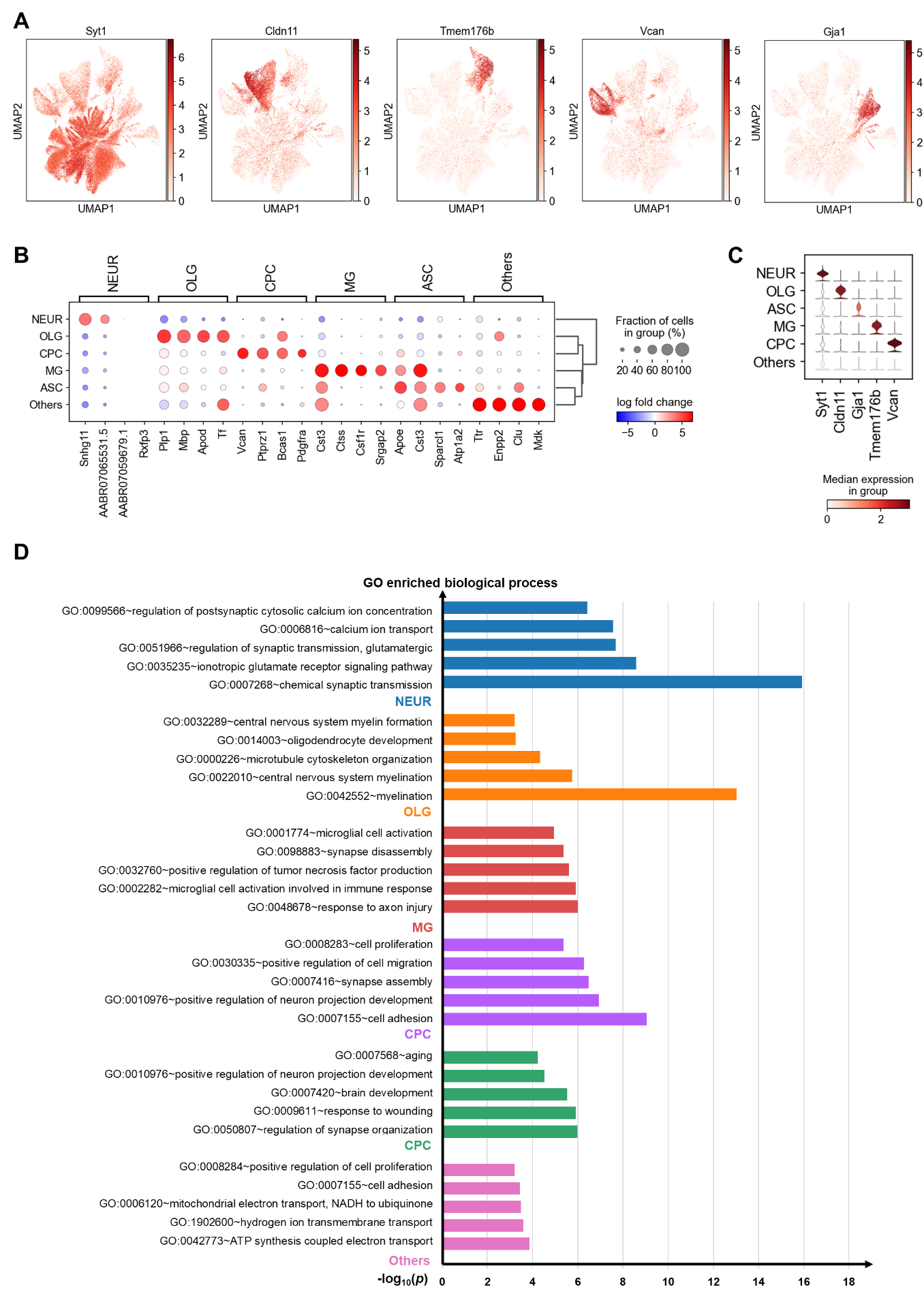
**

**Figure S5. Rats’ hippocampus scRNA-seq data analysis.** **A)** Marker gene expression in each cluster by UMAP analysis. **B)** Clusters are listed on y-axis, showing unbiased gene expression for the top 4 genes per cluster identified by log Fold Change; genes are listed along the x-axis. Dot size reflects percentage of cells in a cluster expressing each gene; dot color reflects expression level. **C)** Violin plots showing the representative marker genes in each cluster. **D)** Functional annotation (DAVID) of each cluster-enriched genes. P values were calculated using DAVID.

**Figure S6**

**
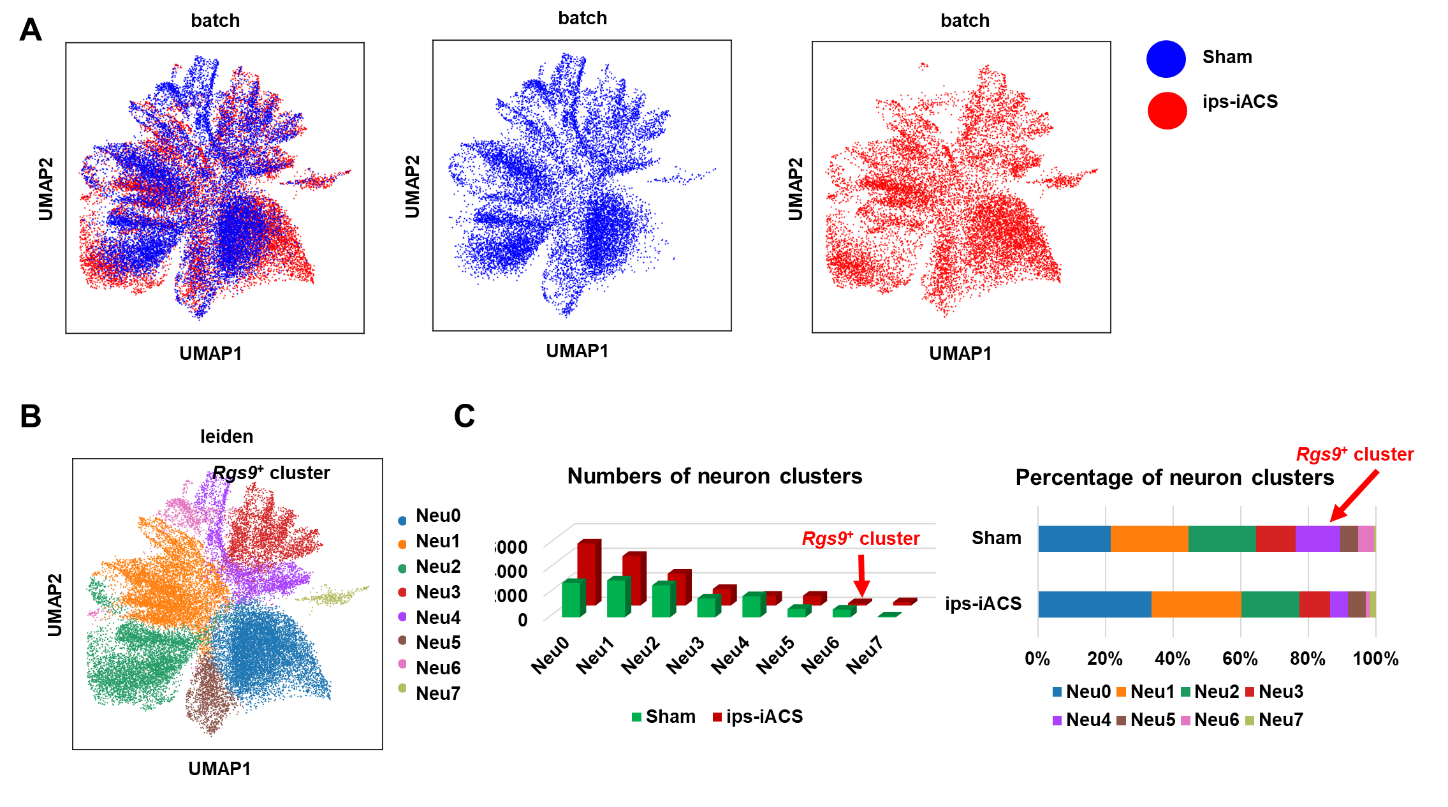
**

**Figure S6. The snRNA-seq data analysis of neurons in the hippocampus.** **A-C)** Uniform Manifold Approximation and Projection (UMAP) embedding of analyzed transcriptomes from sham and ips-iACS cortex neurons annotated by (**A**) treatment of distribution as well as (**B**) neuron subpopulations. **(C)** Neuron numbers and distributions of identified subpopulations.

**Figure S7
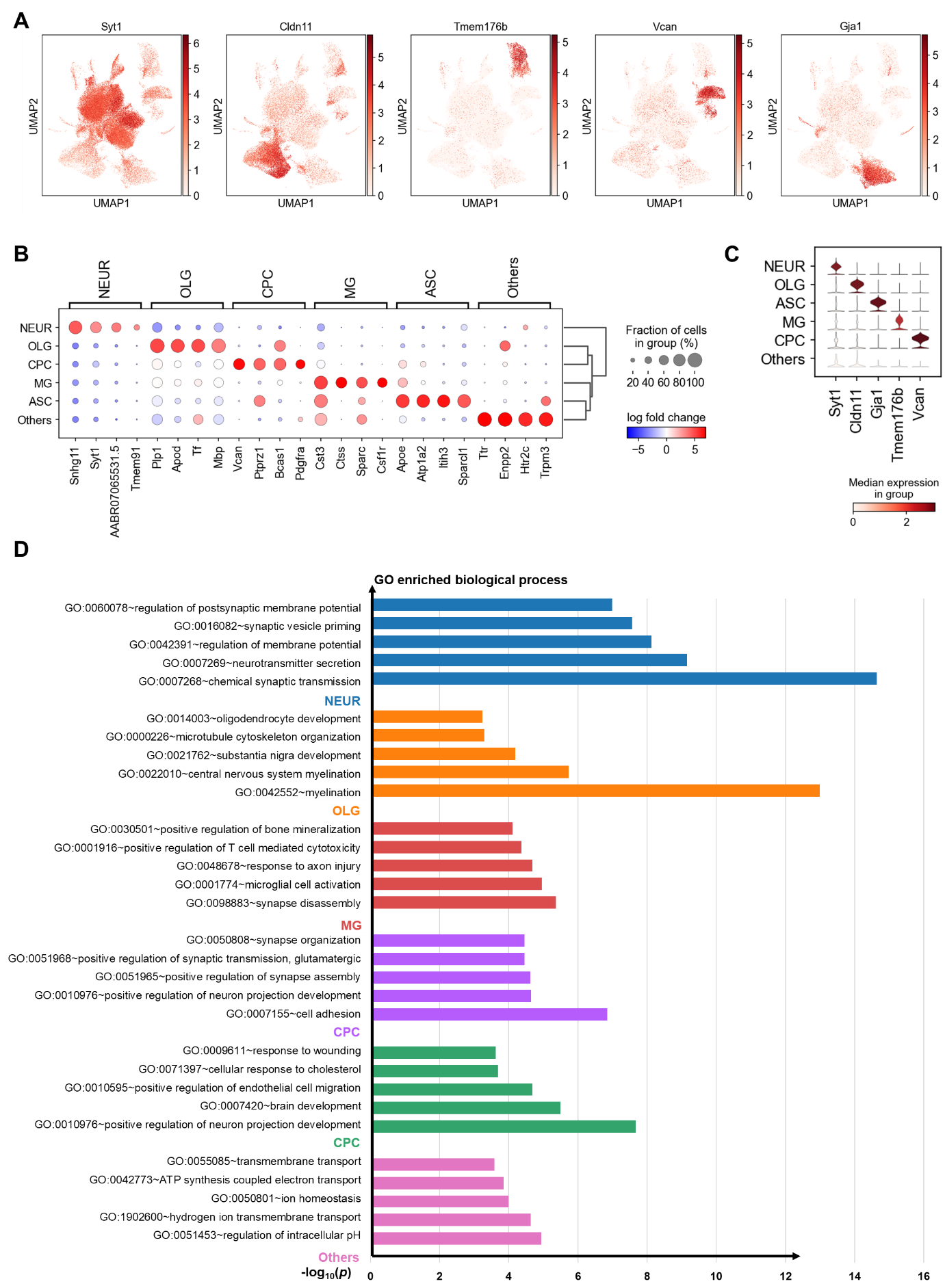
**

**Figure S7. Rats’ thalamus scRNA-seq data analysis.** **A)** Marker gene expression in each cluster by UMAP analysis. **B)** Clusters are listed on y-axis, showing unbiased gene expression for the top 4 genes per cluster identified by log Fold Change; genes are listed along the x-axis. Dot size reflects percentage of cells in a cluster expressing each gene; dot color reflects expression level. **C)** Violin plots showing the representative marker genes in each cluster. **D)** Functional annotation (DAVID) of each cluster-enriched genes. P values were calculated using DAVID.

**Figure S8**

**
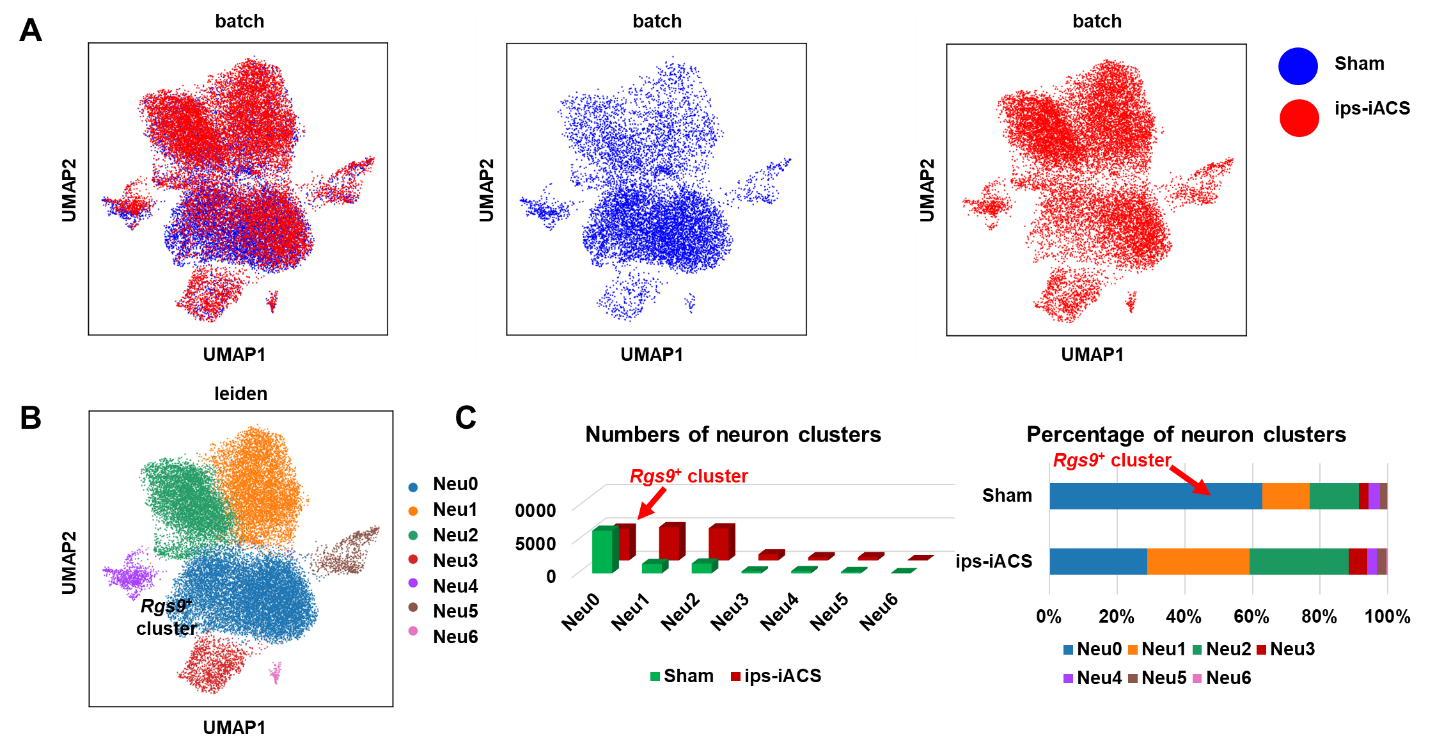
**

**Figure S8. The snRNA-seq data analysis of neurons in the thalamus.** **A-C**) Uniform Manifold Approximation and Projection (UMAP) embedding of analyzed transcriptomes from sham and ips-iACS cortex neurons annotated by (**A**) treatment of distribution as well as (**B**) neuron subpopulations. (**C**) Neuron numbers and distributions of identified subpopulations.

**Figure S9**

**
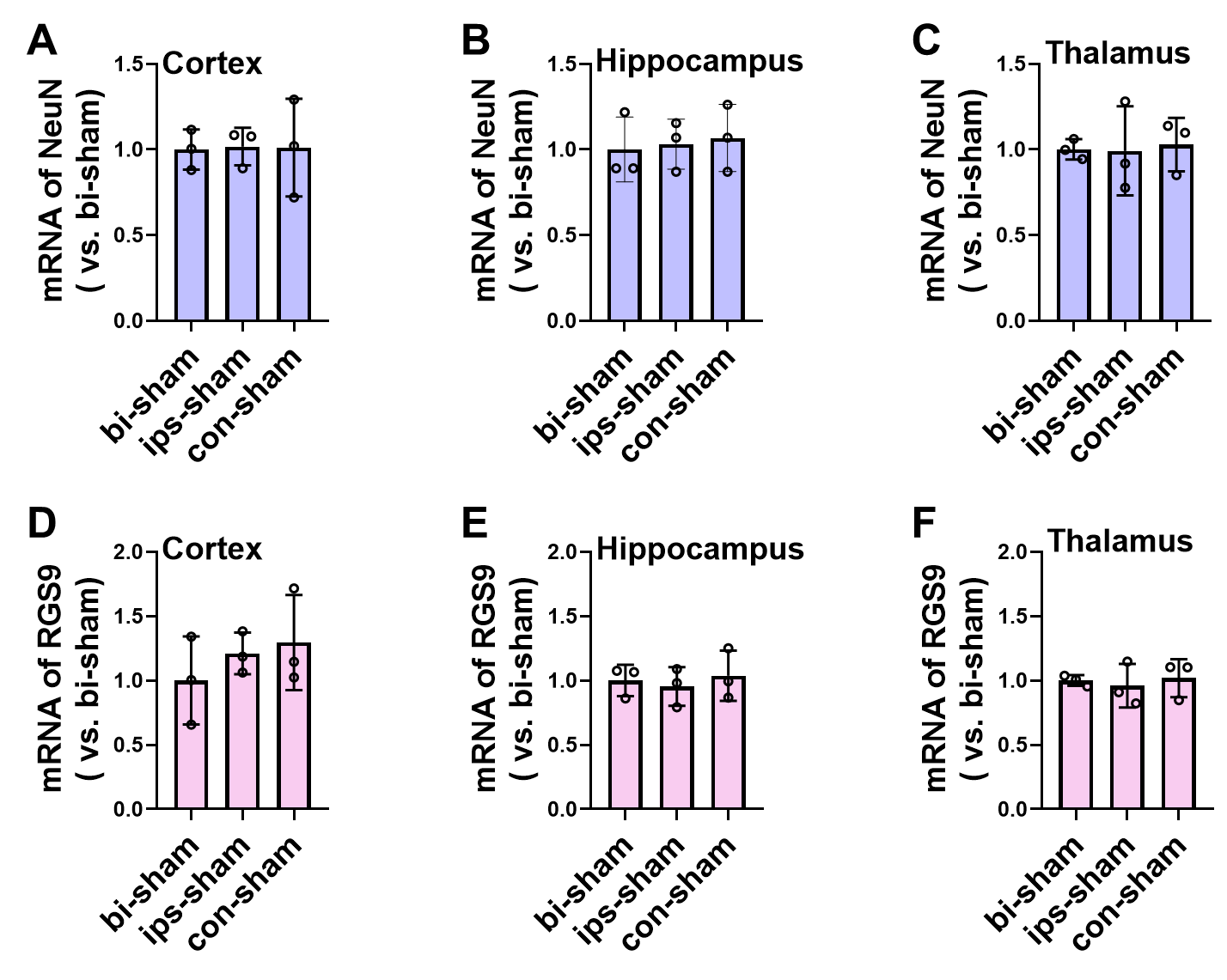
**

**Figure S9. The mRNA expressions of NeuN and *Rgs9* in the cortex, hippocampus and thalamus of the bilateral and unilateral sham rats. A-C)** The NeuN expression in cortex (**A**), hippocampus (**B**) and thalamus (**C**). **D-F)** The *Rgs9* expression in cortex (**A**), hippocampus (**B**) and thalamus (**C**). The mRNA expressions by real-time PCR were shown as mean ± SD, *P < 0.05 was considered as significantly different between each of the three iACS groups and sham. n = 3 rats for each group.
